# Efficient four fragment cloning for the construction of vectors for targeted gene replacement in filamentous fungi

**DOI:** 10.1186/1471-2199-9-70

**Published:** 2008-08-01

**Authors:** Rasmus JN Frandsen, Jens A Andersson, Matilde B Kristensen, Henriette Giese

**Affiliations:** 1 Section of Genetics and Microbiology, Department of Ecology, Faculty of Life Sciences, University of Copenhagen, Thorvaldsensvej 40, DK-1871 Frederiksberg C, Denmark; 2 Department of Systems Biology, Technical University of Denmark, Søltofts Plads, Building 224, room 220, DK-2800 Lyngby, Denmark; 3 Faculty of Agricultural Sciences, University of Aarhus, Blichers Allé 20, DK-8830 Tjele, Denmark

## Abstract

**Background:**

The rapid increase in whole genome fungal sequence information allows large scale functional analyses of target genes. Efficient transformation methods to obtain site-directed gene replacement, targeted over-expression by promoter replacement, in-frame epitope tagging or fusion of coding sequences with fluorescent markers such as GFP are essential for this process. Construction of vectors for these experiments depends on the directional cloning of two homologous recombination sequences on each side of a selection marker gene.

**Results:**

Here, we present a USER Friendly cloning based technique that allows single step cloning of the two required homologous recombination sequences into different sites of a recipient vector. The advantages are: A simple experimental design, free choice of target sequence, few procedures and user convenience. The vectors are intented for *Agrobacterium tumefaciens *and protoplast based transformation technologies. The system has been tested by the construction of vectors for targeted replacement of 17 genes and overexpression of 12 genes in *Fusarium graminearum*. The results show that four fragment vectors can be constructed in a single cloning step with an average efficiency of 84% for gene replacement and 80% for targeted overexpression.

**Conclusion:**

The new vectors designed for USER Friendly cloning provided a fast reliable method to construct vectors for targeted gene manipulations in fungi.

## Background

Filamentous fungi influence modern society primarily as hazardous pathogens and providers of drug leads and enzymes for the biotechnological companies. The wide interest in filamentous fungi has led to the sequencing and annotation of more than 30 fungal genomes, with another 130 on the way . This resource constitutes a huge potential for future advances in the basic understanding and industrial exploitation of fungal biology.

A key limiting factor in future work will be the speed by which site-directed genome modifications, such as gene deletion, promoter replacement, fusion of coding sequences with reporter genes (eg. GFP) or the introduction of epitope tags can be performed and verified. Site directed modifications in filamentous fungi can be carried out by homologous recombination and is typically achieved by introducing a DNA fragment containing two homologous recombination sequences (HRS) flanking a selection marker. The HRS's are identical to the sequences surrounding the target locus in the genome and are typically amplified by PCR. Homologous recombination between the vector DNA and the genome results in a replacement of the target DNA with the selection marker. The length of HRS required to obtain a satisfactory frequency of homologous recombination varies between fungal species. Contrary to *Saccharomyces cerevisiae*, where 30 bp is sufficient, many filamentous fungi require longer HRS [1], eg *Fusarium graminearum *needs 400 bp [2] 1500 bp is reported for *Aspergillus niger *[3] and around 1000 bp for *Neurospora crassa *[4]. The necessity of including sequences in the range of 1 kb [5] makes restriction enzyme and ligation dependent cloning inefficient. Several laboratories have solved this problem by dividing the replacement constructs into two, a technique known as bipartite gene-targeting or split-marker recombination [6-8]. In this technique, the two HRS's are fused with two thirds of either the 3' or 5'end of the selection marker gene, by fusion-PCR [9]. Targeted gene replacement in the fungus is then achieved by a triple homologous recombination between the two PCR fragments and the genomic target, resulting in the formation of a functional selection marker gene and replacement of the targeted locus. The advantage of this technique is that vector construction can be omitted and the PCR products used directly. The technique also increases the efficiency of homologous integration compared to traditional techniques [7]. However, integration of unspecific products generated during the required fusion PCR reactions may give secondary mutations that are difficult to identify. The technique is typically used in combination with protoplast based transformation, which can be difficult or even impossible in some fungal species. Another example of a vector construction system is the one developed for high-throughput knockout of genes in *Neurospora crassa*. Recombinational cloning of the two required HRS with a selection marker gene and a vector backbone is carried out in yeast, followed by PCR amplification of the two HRS and selection marker gene [1]. The amplified DNA is then transformed into *N. crassa *by electroporation, a technique for which no protocols are available for the majority of fungal species.

**Figure 1:**
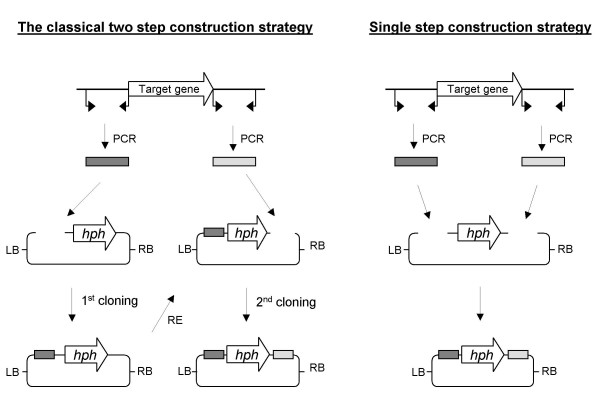
**Strategies for construction of replacement vectors**. On the left, the classical strategy for construction of replacement vectors is shown. It consists of two successive restriction and ligation based cloning steps. On the right the single four fragment USER friendly cloning method is shown. The figures are not drawn to scale. *hph *= *hygromycin phospho-transferase expression cassette (selection marker)*.

The *Agrobacterium tumefaciens *mediated transformation (ATMT) technology [10] has the advantage of being independent of protoplast formation and can be used directly on a wide variety of fungal species and tissue types [11]. ATMT has been widely used for random insertional mutagenesis in eg. *Magnaporthe oryzae *[12], *Leptosphaeria maculans *[13] and *Fusarium oxysporum *[14]. The transformation frequency reported for *F. oxysporum *is between 300–500 transformants per 10^6 ^spores. We have shown that ATMT is an excellent method for site-directed genome modifications in *F. graminearum*, with 200 transformants per 10^6 ^spores, with 60% targeted integrations, using 2 kb HRS's [15]. As ATMT requires both HRS to be present in the same vector, two successive *Escherichia coli *based cloning steps, using four unique cutting enzymes to ensure directionality, are needed. The dependency of unique cutting restriction enzymes complicates the design process and limits the placement of the HRS's [5,15]. A single step vector construction strategy, independent of restriction enzyme sites, would give complete freedom in choice of replacement sites in the genome (Figure 1). This requires an efficient and reliable method for directional four fragment cloning, allowing fusion of the two HRS's on each side of the selection marker gene and to the vector backbone. In recent years new techniques have been developed for highly efficient directional cloning of PCR products into unique restriction sites of vectors, independent of the short overhangs (0–4 bp) generated by standard endonucleases. Examples are the Xi-cloning, In-Fusion cloning, Ligase independent cloning (LIC-PCR), Recombinational cloning and USER Friendly cloning techniques [16-20].

**Figure 2:**
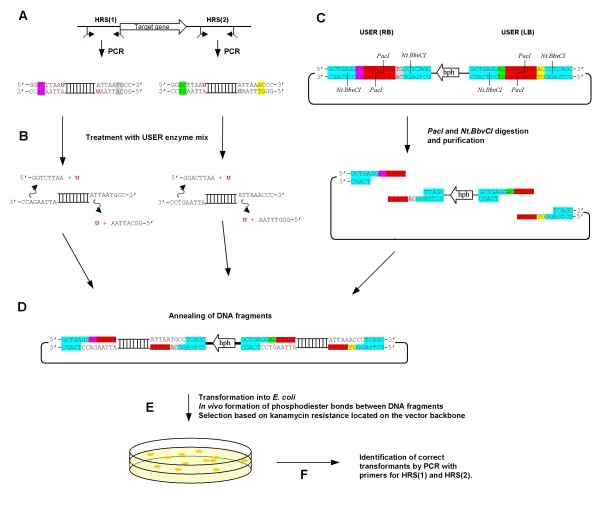
**The USER Friendly cloning strategy for single step construction of replacement vectors**. **A) **Amplification of the two homologous recombination sequences (HRS) with primers that contain 5' deoxyuridine extensions. **B) **Treatment of the PCR amplicons with USER enzyme mix, resulting in the generation of unique 3' single stranded overhangs. The USER enzyme solution is a mixture of Uracil DNA glycosylase and DNA glycosylase-lyase Endo VIII. The Uracil DNA glycosylase recognises the 2'-Deoxyuridine base in the primer portion of the PCR amplicon and excises the uracil nucleobase, resulting in an abasic position [30]. The presence of an abasic site in the DNA permit the DNA glycosylase-lyase Endo VIII to break the phosphodiester backbone at both the 3' and 5' sides of the abasic position, resulting in a single strand break [31]. The resulting short 5' stretch of the original primer then dissociates, leaving the PCR fragment with a 9 bp long 3' single stranded overhang. **C) **Design of the USER vector for targeted gene replacement in fungi, with two unique USER cloning sites (LB and RB). Each of the UCS's consists of a *PacI *site (Red), two *Nt.BbvCI sites *(blue) and two times two unique base pairs (yellow, green, gray and pink) ensuring directional cloning of the inserts. Digestion of the vector results in the generation of two DNA fragments with four unique 9 bp long 3' overhangs. **D) **Mixing and annealing of the two vector DNA fragments and the two inserts. The four unique 3' overhangs ensures correct annealing between the four DNA fragments. **E) **Transformation into *E. coli*, where covalent bonds are formed between the base-paired DNA fragments. **F) **Screening for correct transformants by colony-PCR using the HRS specific primer pairs that were used in step A. The figures are not drawn to scale.

The USER (uracil-specific excision reagent) Friendly cloning technique (New England Biolabs) allows directional cloning of PCR products, independently of restriction enzyme cleavage of the PCR amplicon and DNA ligase for fusion of the amplicon with the vector ends. Instead, vector-specific overhangs (in this paper 9 bp) containing a single 2-deoxyuridine nucleoside, are included in the 5' end of each primer designed to amplify the desired genomic target (Figure 2A). The resulting PCR amplicon (double stranded) is subsequently treated with the USER enzyme mix (Uracil DNA glycosylase and DNA glycosylase-lyase Endo VIII) to create unique 3' single-stranded extensions (Figure 2B). Compatible overhangs (9 bp) in the vector are generated by the combined digestion with a standard restriction enzyme (*PacI*) and a nicking enzyme (*Nt.BbvCI*), where the spacing of the respective recognition sites determines the length of the 3' single stranded overhangs (Figure 2C). Annealing of the digested vector and the USER-treated PCR amplicons enables the formation of a stable recombinant molecule that can be used directly in chemical transformation of *E. coli *without prior ligation (Figure 2D). The DNA pieces are covalently linked by the formation of phosphodiester bonds *in vivo*, most likely catalyzed by the endogenous *E. coli *DNA repair system (Figure 2E).

In the present study, we designed four new vectors, adapted to the USER Friendly cloning technology. Here we show that the technology can be used to generate a large number of targeted gene replacement and overexpression vectors for high throughput functional analysis of fungal genes.

## Methods

### Reagents and enzymes

Restriction enzymes, T4 DNA polymerase, T4 DNA ligase and Calf Intestinal Phosphatase (CIP) and the USER enzyme mix were purchased from New England Biolabs (Ipswich, MA, USA).

Production of amplicons for the USER Friendly cloning reactions were performed with PfuTurbo^® ^C_x _Hotstart DNA polymerase (Stratagene, Cedar Creek, TX, US). The *gpdA *promoter from *Aspergillus nidulans *was amplified with Phusion DNA polymerase (Finnzymes, Espoo, Finland). Screening of *E. coli *transformants was performed with Sigma Taq DNA polymerase (Sigma Aldrich). All PCR reactions were performed using an Eppendorf MasterCycler EP, using the temperature gradient mode for optimization. Sequencing was performed at GATC Biotech (Constance, Germany). Oligos for construction of the USER cloning sites (UCS) were purchased as HPLC purified oligos from Invitrogen (Carlsbad, CA, US). Primers for replacement and overexpression of *Fusarium graminearum *genes were designed based on the *F. graminearum *genome sequence from the Broad Institute  and annotations at the *F. graminearum *Genome DataBase (FGDB) [21] using Vector NTI 10 (Invitrogen). Primers were purchased from Eurofins MWG|operon (Ebersberg, Germany).

If not specified otherwise all enzymes and kits were used as recommended by the manufacturer.

### Preparation of DNA

For production of genomic DNA from *F. graminearum*, the PH-1 wild type strain was grown in liquid YPG medium [22] for three days at 25°C in darkness with stir at 150 rpm. The gDNA was purified following the procedure described by Malz [23]. Plasmid DNA was purified using the Miniprep and Maxiprep kits from QIAgen (Chatsworth, CA, USA). Digested plasmids and PCR products were purified using the GFX purification kit from GE Healthcare. DNA concentrations were measured using a NanoDrop ND-1000 spectrophotometer (Wilmington, DE, US).

### Construction of pAg1-H3E for in locus overexpression

**Figure 3:**
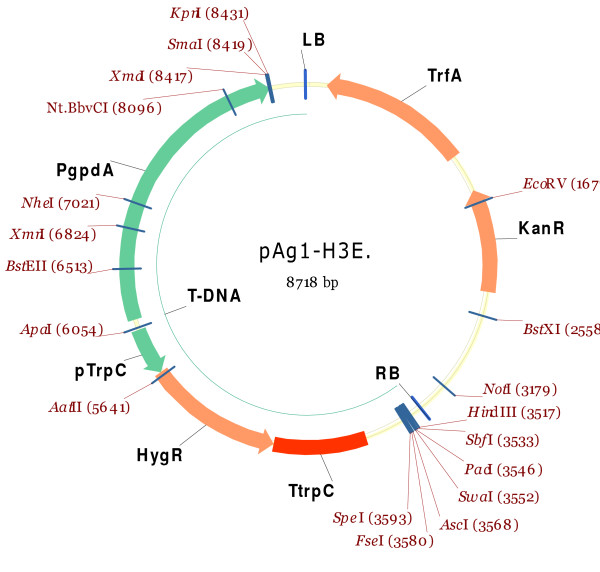
**Expression vector**. The replacement vector pAg1-H3 was converted to an expression vector by introducing the *Aspergillus nidulans gpdA *promoter (PgpdA), into the LB multiple cloning site.

**Figure 4:**
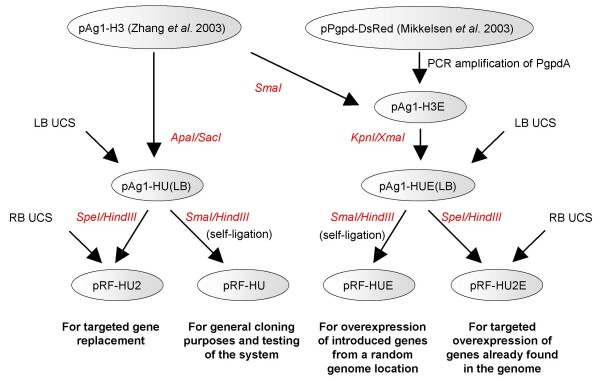
**Construction of USER compatible vectors**. Flow chart for the different components used during construction of the USER compatible vectors. The UCS at the left (LB) and right (RB) borders were ordered as complementary oligomers with additional bases at the ends to make them compatible with the vector ends following digestion with the used restriction enzymes.

To allow overexpression of genes, we constructed a variant of the pAg1-H3 vector [5], in which the *gpdA *promoter from *Aspergillus nidulans *was inserted pointing towards the left border (LB) sequence (Figure 3). The *A. nidulans gpdA *promoter was amplified from pPgpd-DsRed [24] using the primers PgpdA-A1/-A2 [see Additional file 1] and mixed with *SmaI *digested pAg1-H3 (Figure 4). The primers contain 30 bp 5' overhangs that are identical to the blunt *SmaI *generated vector ends of pAg-H3. Directional cloning was carried out according to the Xi-cloning procedure described in [16], with 50 ng *SmaI *digested vector and 150 ng insert, both dephosphorylated and GFX purified. Correct transformants were identified by kanamycin resistance and colony PCR. The orientation of the insert was verified by sequencing with the RF-1 primer.

### Construction of the USER vectors, pRF-HU2 and pRF-HU2E, for targeted replacement

To construct vectors with two unique UCS's flanking the selection marker genes, oligos [see Additional file 1] U2(LB)up/down, U2(RB)up/down and U2(LB)Eup/Edown were mixed to a final concentration of 10 mM each, heated to 90°C for 5 minutes and then cooled to room temperature for annealing of complementary sequences. The ends of the oligos include overhangs that allow directional cloning into the *ApaI*/*SacI *(Left Border) and *SpeI*/*HindIII *(Right Border) sites of the pAg1-H3 vector and into the *KpnI*/*XmaI *(LB) and *SpeI*/*HindIII *(RB) sites of the pAg1-H3E vector.

**Figure 5:**
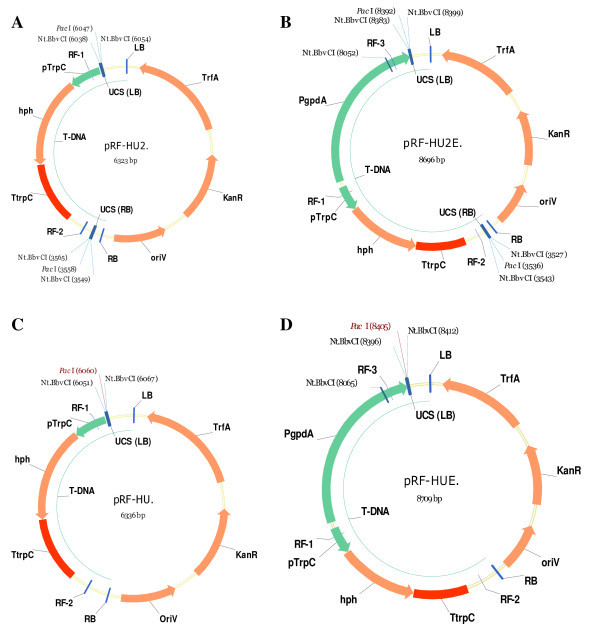
**The constructed vectors for USER Friendly cloning**. The constructed USER vector **(A) **pRF-HU2 (6323 bp), **(B) **pRF-HU2E (8696 bp), **(C) **pRF-HU (6336 bp) and **(D) **pRF-HUE (8709 bp). pRF-HU2 and pRF-HU2E were designed for single step directional cloning of two PCR amplicons to allow targeted gene replacement in filamentous fungi. The pRF-HU and pRF-HUE vectors were designed for construction of vectors for random integration into the fungal genome by non-homologous recombination. The LB UCS is identical in all four vectors, and the RB UCS is identical in the vectors for two fragment cloning. This allows for the reuse of primers with all four vectors. *LB *= left border, *pTrpC *= Tryptophan promoter form *Aspergillus nidulans*, *hph *= hygromycin phosphor transferase, *TtrpC *= Tryptophan terminator from *A. nidulans*, *RB *= right border, *oriV *= origin of replication in *E. coli*, *KanR *= kanamycin resistance, *TrfA *= replication initiation gene (broad-host-range), *PgpdA *= glyceraldehyde-3-phosphate dehydrogenase promoter from *A. nidulans*. Forward primers: RF-1 and RF-3; Reverse primers: RF-2.

**Figure 6:**
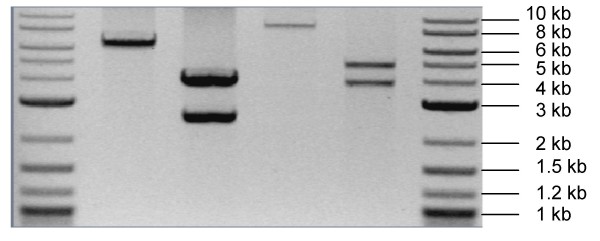
***PacI/Nt.Bbv.CI *digested and GFX purified vectors**. The designed vectors digested with *PacI/Nt.BbvCI *and GFX-purified from solution. Lane 1–6: 2log ladder, pRF-HU, pRF-HU2, pRF-HUE, pRF-HU2E and 2log ladder. Expected sizes of bands: pRF-HU (6336 bp), pRF-HU2 (2489 bp and 3834 bp), pRF-HUE (8709 bp) and pRF-HU2E (3840 bp and 4856 bp).

*ApaI *and *SacI *digested pAg1-H3 vector was GFX-purified and mixed in a 1:5 molar ratio with the double stranded U2(LB)up/down insert and ligated by using T4 DNA ligase. The subsequent transformation of *E. coli *resulted in pAg1-HU(LB). *SpeI *and *HindIII *digested pAg1-HU(LB) was mixed with the double stranded U2(RB)up/down insert and treated as described above. The resulting vector pRF-HU2 (Figure 5 and 6) was sequenced using RF-1 and RF-2 primers.

Similarly, U2(LB)Eup/Edown was ligated into *KpnI *and *XmaI *digested pAg1-H3E, resulting in pAg1-HUE(LB). The resulting vector was then digested with *SpeI *and *HindIII*, mixed with U2(RB)up/down and ligated, resulting in pRF-HU2E (Figure 5 and 6) [see Additional file 2].

### Construction of pRF-HU and pRF-HUE for random integration

For cloning of single PCR amplicons, vectors with only a single UCS were generated. The intermediate vectors (pAg1-HU(LB) and pAg1-HUE(LB)) from the construction of pRF-HU2 and pRF-HU2E, both contain two *PacI *sites, one in the introduced UCS at the LB and one in the RB Multiple Cloning Site (MCS). To allow USER Friendly cloning of single PCR amplicons it was necessary to remove the *PacI *site in the RB MCS. Vectors were digested with *SmaI *and *HindIII *and the resulting ends were polished, using T4 DNA polymerase, and self ligated using T4 DNA ligase. The resulting vectors, pRF-HU and pRF-HUE contain a single USER cloning site at their LB (Figure 5 and 6) [see Additional file 2].

### The USER Friendly cloning reaction and testing of the system

**Figure 7:**
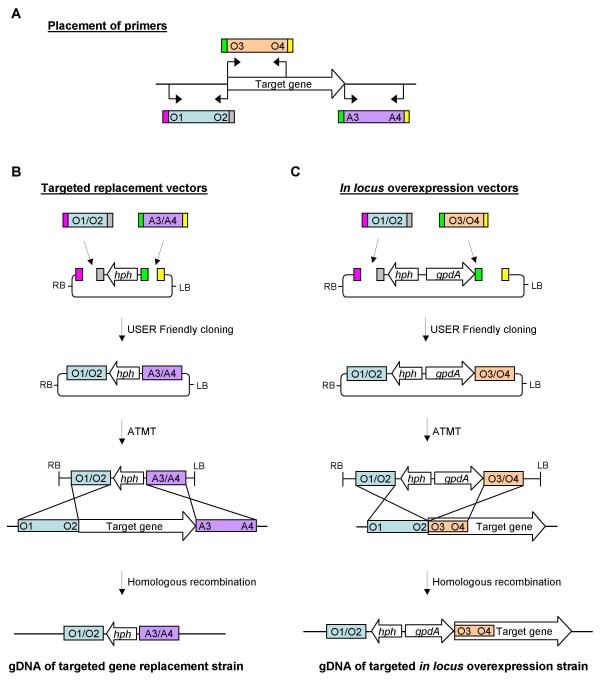
**Placement of the three primer pairs, relative to the target gene**. The designed vectors allow for the reuse of primer pairs for multiple constructs, eg. targeted replacement and *in locus *overexpression. (**A) **Placement of the three primer pairs. (**B**) Construction of the vector for targeted gene replacement and homologous recombination with the genomic target. (**C**) Construction of the vector for *in locus *overexpression and homologous recombination with the genomic target, resulting in insertion of the *gpdA *promoter in front of the coding sequence for the target gene.

The constructed USER vectors were digested with *PacI */*Nt.BbvC IB*, GFX purified and diluted to a concentration ranging from 40–50 ng/μl (Figure 6). The cloning efficiency of the vector system was tested by constructing replacement and *in locus *overexpression vectors for 12 different genes (*PKS1*, *PKS2*, *PKS3 *(*PGL1*), *PKS5*, *PKS6*, *PKS7*, *PKS8*, *PKS9*, *PKS10*, *PKS11, PKS14 *and *PKS15*) and replacement vectors for an additional five genes (*pglJ*, *pglM*, *pglX*, *pglL *and *pglV*) from *Fusarium graminearum*. The test inserts were amplified using three different primer pairs for each of the genes, *geneX*-O1/O2, -O3/O4 and -A3/A4 primer pairs (Figure 7 and [see Additional file 1]) with *F. graminearum *PH-1 gDNA as template.

The designed gene specific primers included 9 bp long 2-Deoxyuridine containing overhangs, O1 = 5'-GGTCTTAA**U**, O2 = 5'-GGCATTAA**U**, O3 = 5'-GGACTTAA**U**, O4 = 5'-GGGTTTAA**U**, A3 = 5'-GGACTTAA**U**, A4 = 5'-GGGTTTAA**U**, which ensured directionality in the cloning reactions (Figure 7). The USER Friendly cloning technique does not require purification of the amplicons prior to cloning and the PCR reactions were therefore used directly, with a DNA concentration ranging from 6 – 15 ng/μl.

For cloning of two PCR amplicons the following components were mixed in a 0.2 ml PCR tube: 1 μl USER enzyme mix (1.0 unit), 10 μl of insert no. 1, 10 μl of insert no. 2 and 200 ng of digested and purified vector in a total reaction volume of 25 μl adjusted with MilliQ water [see Additional file 3]. For single fragment cloning the *geneX*-A3/A4 fragment was mixed with 1 μl USER enzyme mix and 200 ng pRF-HU or pRF-HUE vectors in a reaction volume of 15 μl. The USER reactions were incubated for 20 minutes at 37°C, followed by 20 minutes at 25°C. The entire volume of the USER cloning reactions were used to transform 50 μl of freshly prepared chemical competent *E. coli *cells (10^6 ^cfu./μg DNA), following the transformation guidelines described in [20]. Transformants were selected by adding 25 μg/ml kanamycin to the medium.

The construction of pRF-HU2::*PKS1 *and pRF-HU2E::*PKS1 *vectors were performed in triplicate, using different batches of vector, enzyme and PCR amplicons. Negative controls were performed by replacing the PCR amplicons with MilliQ water.

### Verification of the inserts and their orientation

The resulting *E. coli *transformants for the two *PKS1 *vectors were analysed with the insert specific primer pair to determine the cloning efficiency. All positive transformants were then analysed with primer combinations revealing the orientation of the inserts in the vectors (ex. *PKS1*-O1/RF-2 and RF-1/*PKS1*-O4 or RF-3/*PKS1*-A4). The cloning efficiency was calculated as the number of transformants that contained both inserts in the correct orientation divided by the total number of transformants obtained in the cloning reaction. The size of the vectors was determined by restriction enzyme digestion. The two inserts in the pRF-HU2:*PKS1 *vector were sequenced using the RF-1 and RF-2 primers and the two inserts in the pRF-HU2E::*PKS1 *with the RF-2 and RF-3 primers.

For the other 27 vectors (16 × pRF-HU2 and 11 × pRF-HU2E) that were constructed by USER cloning, ten transformants were picked randomly for each construct and screened with the insert specific primer pairs. The cloning efficiency was calculated as the number of transformants that contained both inserts divided by the number of screened transformants. The size of the individual vectors was evaluated by restriction enzyme digestion, to confirm the cloning efficiency.

## Results and Discussion

The annotation of the sequenced fungal genomes has revealed a plethora of predicted proteins which do not show homology to any previously characterized proteins. In *F. graminearum *PH-1 alone, 8091 out of 13938 (58%) putative genes fall into the categories "Conserved hypothetical protein" or "Hypothetical protein" [21]. Functional characterisation of these putative genes and proteins, to determine their importance for cellular processes related to pathology and synthesis of bioactive secondary metabolites, relies on efficient replacement and overexpression strategies. Implementation of the single step vector construction strategy shown in Figure 1 has the potential to accelerate this research.

The strategy is dependent on a four fragment cloning step, which is laborious and has a very low success rate using classical cloning techniques. The Xi-cloning and In-Fusion technologies allow for four fragment cloning, but had a very low success rate in our experiments (unpublished results). The yeast based construction of gene replacement cassettes for transformation of *N. crassa*, Colot and co-works [1], also allows for efficient four fragment cloning. However, it is dependent on a final PCR based amplification step which increases the risk of unintended mutations in the HRS. These could affect the expression and functionality of the genes surrounding the target locus and sequencing of the integration site would be required to ensure that no unintended mutations have been introduced. The finding that the proofreading PfuTurbo Cx DNA polymerase [25] can amplify templates containing uracil made the USER Friendly cloning technology attractive for our purpose. The use of Proofreading DNA polymerase is essential when making targeted genome modifications in fungi, due to the close spacing of fungal genes [26], which often means that the HRS extends into neighbouring genes or their regulatory sequences. USER Friendly cloning has been used for the directional fusion of multiple PCR amplicons in a single cloning step, but only into a single site of the recipient vector [27]. To test whether the USER Friendly cloning technique would allow the simultaneous cloning of two PCR amplicons into different sites of a recipient vector, vectors with two UCS's were needed. For this purpose we designed two new 26 bp long UCS's with *PacI *and *Nt.Bbv.CI *sites. These were cloned into the ATMT vectors pAg1-H3 and pAg1-H3E, resulting in pRF-HU, pRF-HU2, pRF-HUE and pRF-HU2E (Figure 5), according to the cloning strategy shown in Figure 4. The 26 bp long UCS's are significantly shorter than those utilized in the commercial USER Friendly cloning kit [20] and those reported in [27]. This is an advantage if the vectors have to be modified by the addition of reporter genes or change of the selection marker gene, as shorter 5' overhangs on the primers will be required. Digestion of the new vectors, containing two UCS's, with *PacI *and *Nt.Bbv.CI *results in two fragments with four unique 9 bp long single stranded 3' overhangs, each consisting of seven constant base pairs and two variable bases (Figure 2C). The two variable bases in each overhang provide directionality in the cloning reaction, thereby ensuring correct assembly of the DNA fragments during cloning.

Cloning of a single PCR amplicon was carried out to verify that the designed UCS's were functional. Three independent cloning experiments in which the PCR amplified PKS1-A3/A4 fragment was introduced into the pRF-HU or pRF-HUE vector were performed, resulting in an average cloning efficiency of 95.9% and 95.4% respectively, with an average of 104.7 and 64.7 colonies per transformation (Table 1). The cloning efficiencies for the new and shorter UCS's are very similar to those reported in the commercial system [20].

**Table 1:** Testing of USER Friendly cloning for single step four fragment cloning

			**No. of correct colonies/total no. of colonies**			**Average**
		
**Vector**	**Insert 1**	**Insert 2**	1.	2.	3.	
pRF-HU	PKS1-A1/A2	na	87/91	100/103	114/120	104.7 (95.9%)
pRF-HUE	PKS1-O1/O2	na	59/61	60/63	66/70	64.7 (95.4%)
pRF-HU2	PKS1-A1/A2	PKS1-A3/A4	47/53	52/61	45/55	56.3 (85.2%)
pRF-HU2E	PKS1-O1/O2	PKS1-O3/O4	38/48	39/45	40/49	47.3 (82.4%)

USER cloning: Results from three independent replicates where one or two PCR amplicons are cloned into the designed USER vectors. All transformants were first tested for the presence of the desired insert and then for the orientation of the insert in the vector. All transformants that were positive for inserts also had the correct orientation. The negative control reactions, where no PCR amplicons were added to the reactions, resulted in zero colonies.

The construction of the vectors, by four fragment cloning, for targeted replacement and *in locus *overexpression of *PKS1 *was performed in triplicate and the resulting transformants were screened by PCR. Construction of the targeted replacement vector pRF-HU2::*PKS1 *resulted in an average of 56.3 colonies of which 85.2% tested positive for the two inserts (Table 1). Construction of the overexpression vector pRF-HU2E::*PKS1 *resulted in an average of 47.3 colonies of which 82.4% were correct. All transformants that tested positive for the inserts also displayed the desired orientation in the vector. The negative controls all resulted in zero colonies. Construction of the seventeen different vectors, including *PKS1*, for targeted gene replacement (pRF-HU2) in *F. graminearum *resulted in an average cloning efficiency of 84.1% (+/- 5%) determined by analyses of ten transformants for each of the constructs. The twelve vectors constructed for *in locus *overexpression (pRF-HU2E vectors), including *PKS1*, resulted in an average cloning efficiency of 80.0% (+/- 6%) determined by screening ten transformants for each construct. Based on these results, fusion of two PCR amplicons with two vector fragments by USER Friendly cloning proved to be very efficient as also suggested by the successful fusion of multiple PCR amplicons by Geu-Flores and co-workers [27].

The presence of the same UCS's in the four vectors allow the reuse of primers for different constructs, e.g. replacement and over-expression, thereby reducing the number of primers that are required for the full analysis of a given target gene. This was exploited for the test vectors where the *geneX*-O1/O2 product is reused for both replacement and overexpression vectors. The designed O1/O2 primer pair amplifies the promoter sequence of the respective target genes, the O3/O4 primers amplifies the 5' end of the coding sequences including the start codon, and the A3/A4 primers amplifies the terminator region of the respective genes (Figure 7A). For construction of targeted gene replacement vectors, pRF-HU2 was mixed with *geneX*-O1/O2 and *geneX*-A3/A4. These are required to give homologous recombination with the fungal genome resulting in the replacement of the coding sequence of the target gene (Figure 7B). Vectors for *in locus *over-expression (promoter insertion) were constructed by mixing pRF-HU2E with *geneX*-O1/O2 and *geneX*-O3/O4, so that homologous recombination with the target locus inserts the *A. nidulans gdpA *promoter in front of the start codon of the target gene (Figure 7C).

An additional advantage of the single step construction strategy is that all vector constructs can utilize the same tested vector preparation, which is not the case for the classical two step cloning strategy where cloning of the second insert is dependent on the preparation and digestion of the vector construction in the first cloning step. Besides minimizing the time expenditure for vector construction, the four fragment cloning offers increased flexibility in the choice of selection marker, since the digested vector-backbone can be purified and the hph cassette (Figure 7B and 7C) replaced with other antibiotic selection markers as zeocin or geneticin. This is of high relevance in repeated transformation of hygromycin resistant mutants. Our average vector construction time with the USER Friendly cloning system is three to four days, compared to 14 days with Xi-cloning and 20 days with classical cloning techniques. Furthermore the single step construction strategy only requires a single verification process, whereas two are required for the classical techniques. The constructed vectors are compatible with both *A. tumefaciens *mediated transformation and protoplast based transformation of fungi.

The developed USER Friendly cloning system combined with *A. tumefaciens *mediated transformation, PCR based screening methods [28,15] and quantitative Real-Time PCR to determine the gene copy number [29] can be perfected to give a realistic high throughput system for large scale functional studies of genes in filamentous fungi.

## Conclusion

The constructed pRF-HU2 and pRF-HU2E vectors allow for the simultaneous directional cloning of two inserts in a four fragment assembly with an efficiency of 84.1% and 80.0%, respectively. These results show that the single step four fragment USER Friendly cloning method provides a good alternative to existing vector construction techniques. Vector construction for targeted replacement of genes is reduced to design of two primer pairs, which will permit automation of the experimental design as required for high-throughput knockout projects [1]. The promoters that drive the hygromycin resistance gene in the designed vectors are probably inefficient in basidiomycotes but favourite vectors can easily be converted to USER compatible vectors simply by introducing the two new UCS's into their MCS's.

## Abbreviations

ATMT: *Agrobacterium tumefaciens *mediated transformation; LB: left border of T-DNA (transfer DNA); MCS: multiple cloning site; PCR: polymerase chain reaction; *PKS*: polyketide synthase encoding gene; RB: right border of T-DNA (transfer DNA); UCS: USER cloning site; USER: Uracil Specific Excision Reaction.

## Competing interests

The authors declare that they have no competing interests.

## Authors' contributions

RF conceived the study, performed experiments and analyses, wrote and edited the manuscript. JAA performed validating experiments and analyses. MBK designed and constructed the pAg1-HUE vector. HG contributed to the writing of the manuscript and to the experimental design. All authors read and approved the final manuscript.

## Supplementary Material

Additional File 1 Table S1 – Oligonucleotides used in this study. The primer pairs used in the study. Click here for file

Additional File 2 Vector sequences. The vector sequences in GenBank format. Click here for file

Additional File 3 Protocols for USER Friendly cloning. Click here for file
