# Supplementary material for: Efficient four fragment cloning for the construction of vectors for targeted gene replacement in filamentous fungi
Source: BMC Mol Biol. 2008 Aug 1;9:70. doi: 10.1186/1471-2199-9-70 (PMC2533011; doi:10.1186/1471-2199-9-70)
Supplement: Additional File 1 — Table S1 – Oligonucleotides used in this study. The primer pairs used in the study. [file 1471-2199-9-70-S1.doc]

Supplementary materials

**Table S1 - Oligonucleotides used in this study**

In the *PgpdA* primers the 30 bp long overhangs required for Xi-cloning are in bold. The complementary parts in the oligos used for assembly of the USER Cloning sites (UCS) are in bold. In the primers for testing of the USER vectors the 2-Deoxyuriline bases are highlighted with red, and the two variable positions are highlighted as in figure 2.

| **Name** | **Sequence** | **Product size** |
| --- | --- | --- |
| PgpdA-A1 | 5’- **CCAGTGAATTCGAGCTCGGTACCAAGGCCC**GGGTGATGTCTGCTCAAGCGG | 2302 bp |
| PgpdA-A2 | 5’- **CTTGCGCGCCTAGGCGGCCGTGGCCAGCCC**GAATTCCCTTGTATCTCTAC |
|  |  |  |
| U2(LB)up | 5’- **CGCTGAGGGTTTAATTAAGTCCTCAGCG**GGCC |  |
| U2(LB)down | 5’- **CGCTGAGGACTTAATTAAACCCTCAGCG**AGCT |  |
| U2(RB)up | 5’- CTAGT**GCTGAGGCATTAATTAAGACCTCAGCA** |  |
| U2(RB)down | 5’- AGCTT**GCTGAGGTCTTAATTAATGCCTCAGCA** |  |
| U2(LB)Eup | 5’- **CGCTGAGGGTTTAATTAAGTCCTCAGCC** |  |
| U2(LB)Edown | 5’- CCGGG**GCTGAGGACTTAATTAAACCCTCAGC**GGTAC |  |
|  |  |  |
| RF-1 | 5’- AAATTTTGTGCTCACCGCCTGGAC |  |
| RF-2 | 5’- TCTCCTTGCATGCACCATTCCTTG |  |
| RF-3 | 5’- TTGCGTCAGTCCAACATTTGTTGCCA |  |
|  |  |  |
| PKS1-O1 | 5’- GGTCTTAAUAGAGGGACTAGCCAACCAAAGATAAGA | 1509 bp |
| PKS1-O2 | 5’- GGCATTAAUGTTTTCCTCTACATAATATCTTCCATCC |
| PKS1-O3 | 5’- GGACTTAAUGTTTTTCCCGAAGTCCCCTTG | 1400 bp |
| PKS1-O4 | 5’- GGGTTTAAUCTTCTGTATGGCCAATCTAGCATGTTC |
| PKS1-A3 | 5’- GGACTTAAUAGAGATGCGGAACTGGCTCCTAGA | 1207 bp |
| PKS1-A4 | 5’- GGGTTTAAUTGGCTTCGTTATGCGGGATACTG |
|  |  |  |
| PKS2-O1 | 5’- GGTCTTAAUGGCATTACTAGGACGACACAGATCAAG | 1401 bp |
| PKS2-O2 | 5’- GGCATTAAUCGTTCCAAAAGGGCAAACTCTACA |
| PKS2-O3 | 5’- GGACTTAAUGTACAATCATCAAGGCAAAAACAACAAG | 1058 bp |
| PKS2-O4 | 5’- GGGTTTAAUCTTGTTTGTAAACATGACGGATCAGCT |
| PKS2-A3 | 5’- GGACTTAAUTTAGGGAGCCATTTCAGTTTAGAATAGTTG | 1400 bp |
| PKS2-A4 | 5’- GGGTTTAAUCGTTCCAAAAGGGCAAACTCTACA |
|  |  |  |
| PKS3-O1 | 5’- GGTCTTAAUCGAGGTGGGATAAAAATACTTACATC | 1294 bp |
| PKS3-O2 | 5’- GGCATTAAUAACAAAGGTGTTGAATCTTGTAAAG |
| PKS3-O3 | 5’- GGACTTAAUATGGCTTCCCATATTAAGCTGTATCTC | 1222 bp |
| PKS3-O4 | 5’- GGGTTTAAUGCAACGATAGCAAGCTTTGGCTT |
| PKS3-A3 | 5’- GGACTTAAUTGGACACCTGTCAACGACATCGCTGAT | 1889 bp |
| PKS3-A4 | 5’- GGGTTTAAUTAGGAGGTCAGCCTTTGTGATGCCACC |
|  |  |  |
| PKS5-O1 | 5’- GGTCTTAAUGGATTCCACTGTCCAAAAGATCAAAA | 1400 bp |
| PKS5-O2 | 5’- GGCATTAAUATTGGCACAATAGTTGGACAGTTTCTG |
| PKS5-O3 | 5’- GGACTTAAUGAGCCCCCCAGCCCCAATTG | 1568 bp |
| PKS5-O4 | 5’- GGGTTTAAUGGGCCGTTCTGTGGCGTAGGTAACT |
| PKS5-A3 | 5’- GGACTTAAUGGCTTTGGATAGAGCAGCCGAGATTGTGA | 1400 bp |
| PKS5-A4 | 5’- GGGTTTAAUCCTGGGCCCCATGCTGAATGCAGTA |
|  |  |  |
| PKS6-O1 | 5’- GGTCTTAAUAATAGCCTGTCAGACATGCATCCAG | 1412 bp |
| PKS6-O2 | 5’- GGCATTAAUGGTGTTCAGGGAGATTTAGAATAAGA |
| PKS6-O3 | 5’- GGACTTAAUGGGCTCACTTAGCGCCGTCC | 1202 bp |
| PKS6-O4 | 5’- GGGTTTAAUGCCTCGACATAGTCCGCATACGGTG |
| PKS6-A3 | 5’- GGACTTAAUGGAGGAAGAACAGGTTGATGTAGTGAAG | 1411 bp |
| PKS6-A4 | 5’- GGGTTTAAUCTGTCAGTTGTTAGTGGCTATGTAACTATGTCT |
|  |  |  |
| PKS7-O1 | 5’- GGTCTTAAUGTGGACAAACGTTTATGGGCTGTCAG | 1413 bp |
| PKS7-O2 | 5’- GGCATTAAUGGCGCCAAAAGGCAGACCCAAA |
| PKS7-O3 | 5’- GGACTTAAUGGAAGACGACATCGCTGTCGTG | 1300 bp |
| PKS7-O4 | 5’- GGGTTTAAUCTTTAAGCTCTCGCCAGTCTCCG |
| PKS7-A3 | 5’- GGACTTAAUTGGTTTTGGAGTAGAGGCCAACTAGAGATGT | 1400 bp |
| PKS7-A4 | 5’- GGGTTTAAUTCACAGCTGGCCGATGAAGTTT |
|  |  |  |
| PKS8-O1 | 5’- GGTCTTAAUGGATCTAATTCTAGACCCCTGCATGG | 1424 bp |
| PKS8-O2 | 5’- GGCATTAAUGTGTGATACAGGATGAACTGAACAGAACA |
| PKS8-O3 | 5’- GGACTTAAUGCCTTCTCAAATTCAACCATGGC | 1400 bp |
| PKS8-O4 | 5’- GGGTTTAAUATCGTTAGCTATACATGATGGTATGTGTGG |
| PKS8-A3 | 5’- GGACTTAAUTGATTGTGTGAGAGATAGTTGTTGGATCTT | 1406 bp |
| PKS8-A4 | 5’- GGGTTTAAUAACATCTACCCTTTAGTAATCCAACACACTG |
|  |  |  |
| PKS9-O1 | 5’- GGTCTTAAUTTATGTTTGCGCAGAGTCCCAACA | 1437 bp |
| PKS9-O2 | 5’- GGCATTAAUGGCAATTGGTTCGTTGGTCGGT |
| PKS9-O3 | 5’- GGACTTAAUGCAAGGACCGACCAACGAACCAATTG | 1540 bp |
| PKS9-O4 | 5’- GGGTTTAAUCTGAGGGGCCTGAGAGTATGCTTCTAGAATGG |
| PKS9-A3 | 5’- GGACTTAAUTAAGGAAAGACAAGAGCAGGAAACAAGA | 1401 bp |
| PKS9-A4 | 5’- GGGTTTAAUCCTCATTCGGGATATCAACATTCTTTT |
|  |  |  |
| PKS10-O1 | 5’- GGTCTTAAUACGGCTGCGAGTAATCTCAAACAGA | 1400 bp |
| PKS10-O2 | 5’- GGCATTAAUGCGTGTAATTCTGATAGTCAGAAGATGTTGAAG |
| PKS10-O3 | 5’- GGACTTAAUGTCGAGTCAATCTTTCCCAAAAG | 1404 bp |
| PKS10-O4 | 5’- GGGTTTAAUGATAACTTCATTGACCGAGCTTTCTGA |
| PKS10-A3 | 5’- GGACTTAAUCCTGGCTTCGAACGGGTAGTTC | 1400 bp |
| PKS10-A4 | 5’- GGGTTTAAUAGCAGCATCAAAGCCTTGGGTAAG |
|  |  |  |
| PKS11-O1 | 5’- GGTCTTAAUTTCATCTACTTCCCCACCTTGTCCT | 1401 bp |
| PKS11-O2 | 5’- GGCATTAAUTCGTTGATCATGGTTGCTTAGTTATGG |
| PKS11-O3 | 5’- GGACTTAAUGATCAACGACACTGGCCCAGAG | 1402 bp |
| PKS11-O4 | 5’- GGGTTTAAUGCTCATGCTTTGATGAAGGCCA |
| PKS11-A3 | 5’- GGACTTAAUATGTATTTGGCAGCACATAGGTACTGTCT | 1400 bp |
| PKS11-A4 | 5’- GGGTTTAAUAAGGGAAAGGCTTGAAGGTGTTCTT |
|  |  |  |
| PKS14-O1 | 5’- GGGTTTAAUACCGGTAGTCGCCCTCTGAGATGAAG | 1575 bp |
| PKS14-O2 | 5’- GGACTTAAUGGCCGCGCGTCGGGTAGTACTGTT |
| PKS14-O3 | 5’- GGCATTAAUGATGGACGTAGGCTGGTTGGAT | 1600 bp |
| PKS14-O4 | 5’- GGTCTTAAUGTTGTATCGACGCCCTTGACGT |
| PKS14-A3 | 5’- GGCATTAAUCGCAAATATACTGATATTGCCTACAGGTCA | 1522 bp |
| PKS14-A4 | 5’- GGTCTTAAUCGTTATTTACATACAAACCATCTCGCTGTC |
|  |  |  |
| PKS15-O1 | 5’- GGTCTTAAUCCATGATTTGGCGCCTCAGTT | 1216 bp |
| PKS15-O2 | 5’- GGCATTAAUGGTGAATCTGTAGCCATGGTTCTGA |
| PKS15-O3 | 5’- GGACTTAAUGGCTACAGATTCACCCTCATTGCTAA | 1407 bp |
| PKS15-O4 | 5’- GGGTTTAAUAGGATCCATATGCTCGGCTTCG |
| PKS15-A3 | 5’- GGACTTAAUGATTAATCACGGCATGGCGGT | 1400 bp |
| PKS15-A4 | 5’- GGGTTTAAUCGATGATTGGAATGTACTATTGTGGGAC |
|  |  |  |
| pglJ-A1 | 5´- GGGTTTAAUAGCTTGGCTGAGCTTGTGTTAGTGA | 1000 bp |
| pglJ-A2 | 5’- GGACTTAAUCATCTTGGGTTAGGTTATGTTGTATGCA |
| pglJ-A3 | 5’- GGCATTAAUTAGATTGGGGTAGATTGGGGTAGATTTG | 1003 bp |
| pglJ-A4 | 5’- GGTCTTAAUACTGGCAGCTCAAGTTCAGAGATCC |
|  |  |  |
| pglM-A1 | 5´- GGGTTTAAUTCATGTTAGCCACAGCATTTCTCAATC | 1000 bp |
| pglM-A2 | 5’- GGACTTAAUTAAGCGAGTTTAGGGGAGTGTTAGGG |
| pglM-A3 | 5’- GGCATTAAUCATATCTTGGGCATGCGTTTTCTC | 1000 bp |
| pglM-A4 | 5’- GGTCTTAAUGCGGTTTATTATGTGCAGAATCTCAAA |
|  |  |  |
| pglL-A1 | 5´- GGGTTTAAUACAACACCCTGCAGGACTTGGC | 1000 bp |
| pglL-A2 | 5’- GGACTTAAUTAAAGAGCAAAAAGGTGAAAATTATACG |
| pglL-A3 | 5’- GGCATTAAUCATTGTGATTTTTTTGAATGATATC | 1000 bp |
| pglL-A4 | 5’- GGTCTTAAUAGTAGTCACTACTCTAGATGATGAAC |
|  |  |  |
| pglX-A1 | 5´- GGGTTTAAUAATCTCAAGGAAACTGGCCT | 1000 bp |
| pglX-A2 | 5’- GGACTTAAUTGATTGAAATACTTTTCAATAATAT |
| pglX-A3 | 5’- GGCATTAAUAGAGCTGTTCTGTTCGACAGACGATAA | 1039 bp |
| pglX-A4 | 5’- GGTCTTAAUTGAGGCTTCTTCATTCACCGTCG |
|  |  |  |
| pglV-A1 | 5´- GGGTTTAAUTCATACACTCCTTCGCCAACCTCA | 1000 bp |
| pglV-A2 | 5’- GGACTTAAUTAGAACGGAGAGACTGGTCTCCGA |
| pglV-A3 | 5’- GGCATTAAUCATTTTGATGATTTGAAGTTTCAATTG | 1028 bp |
| pglV-A4 | 5’- GGTCTTAAUCGCAATTATCTTGACATACAATATTGGC |
