# Supplementary material for: Efficient four fragment cloning for the construction of vectors for targeted gene replacement in filamentous fungi
Source: BMC Mol Biol. 2008 Aug 1;9:70. doi: 10.1186/1471-2199-9-70 (PMC2533011; doi:10.1186/1471-2199-9-70)
Supplement: Additional File 2 — Vector sequences. The vector sequences in GenBank format. [file 1471-2199-9-70-S2.doc]

**Supplementary information for the article: “Efficient four fragment cloning for the construction of vectors for targeted gene replacement in filamentous fungi”**

Rasmus J.N. Frandsen (raf@life.ku.dk), Jens A. Andersson, Matilde B. Kristensen and Henriette Giese

Section of Genetics and Microbiology, Department of Ecology, Faculty of Life Sciences, University of Copenhagen, Thorvaldsensvej 40, DK-1871 Frederiksberg C, Denmark.

Vector sequences in full GenBank format:

**pRF-HU**

LOCUS pRF-HU 6336 bp DNA circular 11-JUL-2007

DEFINITION Vector for easy cloning and ectopic integration of marker genes in filamentous fungi.

SOURCE

ORGANISM

COMMENT The vector can be used for ectopic integration of marker genes into the

genome of filamentous fungi.

The vector has been designed for the directional cloning of a single PCR

amplicon, utilizing the USER friendly cloning technology from NEB.

Add the following 5' overhangs to the primers for directional cloning of the

amplicon into the vector:

Gene-A3 (LB forward) 5´- GGACTTAAU - Forward primer sequence

Gene-A4 (LB reverse) 5'- GGGTTTAAU - Reverse primer sequence

Digestion of vector prior to cloning:

1. Digest overnight with PacI

2. Digest for 2 hours with Nt.BbvCI (nicking enzyme)

3. Purify the vector DNA

Transformation of the vector into the fungi:

The vector is originally designed for Agrobacterium tumefaciens mediated

transformation, but can also be used for protoplast transformation.

COMMENT AUTHORNAME|Rasmus J.N. Frandsen|

COMMENT AUTHOREML|raf@life.ku.dk|

COMMENT REPLTYPE|Plasmid

COMMENT EXTCHREPL|Bacteria

FEATURES Location/Qualifiers

promoter 313..677

/label=pTrpC

misc_structure 2871..2893

/label=RB

terminator 1727..2443

/label=TtrpC

CDS 678..1723

/label=hph

CDS 3945..4740

/label=KanR

CDS 5039..6188

/label=TrfA

misc_structure 6315..6336

/label=LB

misc_feature 2630..2653

/label=RF-2

misc_feature 266..291

/label=UCS LB

primer_bind complement(400..423)

/label=RF-1

rep_origin 3041..3658

/label=OriV

BASE COUNT 1497 a 1616 c 1796 g 1427 t

ORIGIN

1 caaattgacg cttagacaac ttaataacac attgcggacg tttttaatgt actggggtgg

61 tttttctttt caccagtgag acgggcaaca gcggcgccat tcgccattca ggctgcgcaa

121 ctgttgggaa gggcgatcgg tgcgggcctc ttcgctatta cgccagctgg cgaaaggggg

181 atgtgctgca aggcgattaa gttgggtaac gccagggttt tcccagtcac gacgttgtaa

241 aacgacggcc agtgaattcg agctcgctga gggtttaatt aagtcctcag cgggcccatc

301 gatgatcagg cctcgacaga agatgatatt gaaggagcac tttttgggct tggctggagc

361 tagtggaggt caacaatgaa tgcctatttt ggtttagtcg tccaggcggt gagcacaaaa

421 tttgtgtcgt ttgacaagat ggttcattta ggcaactggt cagatcagcc ccacttgtag

481 cagtagcggc ggcgctcgaa gtgtgactct tattagcaga caggaacgag gacattatta

541 tcatctgctg cttggtgcac gataacttgg tgcgtttgtc aagcaaggta agtgaacgac

601 ccggtcatac cttcttaagt tcgcccttcc tccctttatt tcagattcaa tctgacttac

661 ctattctacc caagcatcga tatgaaaaag cctgaactca ccgcgacgtc tgtcgagaag

721 tttctgatcg aaaagttcga cagcgtctcc gacctgatgc agctctcgga gggcgaagaa

781 tctcgtgctt tcagcttcga tgtaggaggg cgtggatatg tcctgcgggt aaatagctgc

841 gccgatggtt tctacaaaga tcgttatgtt tatcggcact ttgcatcggc cgcgctcccg

901 attccggaag tgcttgacat tggggaattc agcgagagcc tgacctattg catctcccgc

961 cgtgcacagg gtgtcacgtt gcaagacctg cctgaaaccg aactgcccgc tgttctgcag

1021 ccggtcgcgg aggccatgga tgcgatcgct gcggccgatc ttagccagac gagcgggttc

1081 ggcccattcg gaccgcaagg aatcggtcaa tacactacat ggcgtgattt catatgcgcg

1141 attgctgatc cccatgtgta tcactggcaa actgtgatgg acgacaccgt cagtgcgtcc

1201 gtcgcgcagg ctctcgatga gctgatgctt tgggccgagg actgccccga agtccggcac

1261 ctcgtgcacg cggatttcgg ctccaacaat gtcctgacgg acaatggccg cataacagcg

1321 gtcattgact ggagcgaggc gatgttcggg gattcccaat acgaggtcgc caacatcttc

1381 ttctggaggc cgtggttggc ttgtatggag cagcagacgc gctacttcga gcggaggcat

1441 ccggagcttg caggatcgcc gcggctccgg gcgtatatgc tccgcattgg tcttgaccaa

1501 ctctatcaga gcttggttga cggcaatttc gatgatgcag cttgggcgca gggtcgatgc

1561 gacgcaatcg tccgatccgg agccgggact gtcgggcgta cacaaatcgc ccgcagaagc

1621 gcggccgtct ggaccgatgg ctgtgtagaa gtactcgccg atagtggaaa ccgacgcccc

1681 agcactcgtc cgagggcaaa ggaatagagt agatgccgac cgggatccac ttaacgttac

1741 tgaaatcatc aaacagcttg acgaatctgg atataagatc gttggtgtcg atgtcagctc

1801 cggagttgag acaaatggtg ttcaggatct cgataagata cgttcatttg tccaagcagc

1861 aaagagtgcc ttctagtgat ttaatagctc catgtcaaca agaataaaac gcgtttcggg

1921 tttacctctt ccagatacag ctcatctgca atgcattaat gcattggacc tcgcaaccct

1981 agtacgccct tcaggctccg gcgaagcaga agaatagctt agcagagtct attttcattt

2041 tcgggagacg agatcaagca gatcaacggt cgtcaagaga cctacgagac tgaggaatcc

2101 gctcttggct ccacgcgact atatatttgt ctctaattgt actttgacat gctcctcttc

2161 tttactctga tagcttgact atgaaaattc cgtcaccagc ccctgggttc gcaaagataa

2221 ttgcactgtt tcttccttga actctcaagc ctacaggaca cacattcatc gtaggtataa

2281 acctcgaaaa tcattcctac taagatgggt atacaatagt aaccatggtt gcctagtgaa

2341 tgctccgtaa cacccaatac gccggccgaa acttttttac aactctccta tgagtcgttt

2401 acccagaatg cacaggtaca cttgtttaga ggtaatcctt ctttctagag gatcctctac

2461 gccggacgca tcgtggccgg catcaccggc gccacaggtg cggttgctgg cgcctatatc

2521 gccgacatca ccgatgggga agatcgggct cgccacttcg ggctcatgag cgcttgtttc

2581 ggcgtgggta tggtggcagg ccccgtggcc gggggactgt tgggcgccat ctccttgcat

2641 gcaccattcc ttgcggcggc ggtgctcaac ggcctcaacc tactactggg ctgcttccta

2701 atgcaggagt cgcataaggg agagcgtcga gatctagagg atcccccgac tagtgcgcga

2761 tcgcggccgg ccggcgcgcc gtttaaacgg atttagcttc gtgactccct taattctccg

2821 ctcatgatca gattgtcgtt tcccgccttc agtttaaact atcagtgttt gacaggatat

2881 attggcgggt aaacctaaga gaaaagagcg tttattagaa taatcggata tttaaaaggg

2941 cgtgaaaagg tttatccgtt cgtccatttg tttgttcatg ccaaccacag ggttccagat

3001 ccgacgagca aggcaagacc gagcgccttt gcgacgctca ccgggctggt tgccctcgcc

3061 gctgggctgg cggccgtcta tggccctgca aacgcgccag aaacgccgtc gaagccgtgt

3121 gcgagacacc gcggccgccg gcgttgtgga tacctcgcgg aaaacttggc cctcactgac

3181 agatgagggg cggacgttga cacttgaggg gccgactcac ccggcgcggc gttgacagat

3241 gaggggcagg ctcgatttcg gccggcgacg tggagctggc cagcctcgca aatcggcgaa

3301 aacgcctgat tttacgcgag tttcccacag atgatgtgga caagcctggg gataagtgcc

3361 ctgcggtatt gacacttgag gggcgcgact actgacagat gaggggcgcg atccttgaca

3421 cttgaggggc agagtgctga cagatgaggg gcgcacctat tgacatttga ggggctgtcc

3481 acaggcagaa aatccagcat ttgcaagggt ttccgcccgt ttttcggcca ccgctaacct

3541 gtcttttaac ctgcttttaa accaatattt ataaaccttg tttttaacca gggctgcgcc

3601 ctgtgcgcgt gaccgcgcac gccgaagggg ggtgcccccc cttctcgaac cctcccggcc

3661 cgctaacgcg ggcctcccat ccccccaggc gtacgccact ggagcacctc aaaaacacca

3721 tcatacacta aatcagtaag ttggcagcat cacccataat tgtggtttca aaatcggctc

3781 cgtcgatact atgttatacg ccaactttga aaacaacttt gaaaaagctg ttttctggta

3841 tttaaggttt tagaatgcaa ggaacagtga attggagttc gtcttgttat aattagcttc

3901 ttggggtatc tttaaatact gtagaaaaga ggaaggaaat aataaatggc taaaatgaga

3961 atatcaccgg aattgaaaaa actgatcgaa aaataccgct gcgtaaaaga tacggaagga

4021 atgtctcctg ctaaggtata taagctggtg ggagaaaatg aaaacctata tttaaaaatg

4081 acggacagcc ggtataaagg gaccacctat gatgtggaac gggaaaagga catgatgcta

4141 tggctggaag gaaagctgcc tgttccaaag gtcctgcact ttgaacggca tgatggctgg

4201 agcaatctgc tcatgagtga ggccgatggc gtcctttgct cggaagagta tgaagatgaa

4261 caaagccctg aaaagattat cgagctgtat gcggagtgca tcaggctctt tcactccatc

4321 gacatatcgg attgtcccta tacgaatagc ttagacagcc gcttagccga attggattac

4381 ttactgaata acgatctggc cgatgtggat tgcgaaaact gggaagaaga cactccattt

4441 aaagatccgc gcgagctgta tgatttttta aagacggaaa agcccgaaga ggaacttgtc

4501 ttttcccacg gcgacctggg agacagcaac atctttgtga aagatggcaa agtaagtggc

4561 tttattgatc ttgggagaag cggcagggcg gacaagtggt atgacattgc cttctgcgtc

4621 cggtcgatca gggaggatat cggggaagaa cagtatgtcg agctattttt tgacttactg

4681 gggatcaagc ctgattggga gaaaataaaa tattatattt tactggatga attgttttag

4741 tacctagatg tggcgcaacg atgccggcga caagcaggag cgcaccgact tcttccgcat

4801 caagtgtttt ggctctcagg ccgaggccca cggcaagtat ttgggcaagg ggtcgctggt

4861 attcgtgcag ggcaagattc ggaataccaa gtacgagaag gacggccaga cggtctacgg

4921 gaccgacttc attgccgata aggtggatta tctggacacc aaggcaccag gcgggtcaaa

4981 tcaggaataa gggcacattg ccccggcgtg agtcggggca atcccgcaag gagggtgaat

5041 gaatcggacg tttgaccgga aggcatacag gcaagaactg atcgacgcgg ggttttccgc

5101 cgaggatgcc gaaaccatcg caagccgcac cgtcatgcgt gcgccccgcg aaaccttcca

5161 gtccgtcggc tcgatggtcc agcaagctac ggccaagatc gagcgcgaca gcgtgcaact

5221 ggctccccct gccctgcccg cgccatcggc cgccgtggag cgttcgcgtc gtctcgaaca

5281 ggaggcggca ggtttggcga agtcgatgac catcgacacg cgaggaacta tgacgaccaa

5341 gaagcgaaaa accgccggcg aggacctggc aaaacaggtc agcgaggcca agcaggccgc

5401 gttgctgaaa cacacgaagc agcagatcaa ggaaatgcag ctttccttgt tcgatattgc

5461 gccgtggccg gacacgatgc gagcgatgcc aaacgacacg gcccgctctg ccctgttcac

5521 cacgcgcaac aagaaaatcc cgcgcgaggc gctgcaaaac aaggtcattt tccacgtcaa

5581 caaggacgtg aagatcacct acaccggcgt cgagctgcgg gccgacgatg acgaactggt

5641 gtggcagcag gtgttggagt acgcgaagcg cacccctatc ggcgagccga tcaccttcac

5701 gttctacgag ctttgccagg acctgggctg gtcgatcaat ggccggtatt acacgaaggc

5761 cgaggaatgc ctgtcgcgcc tacaggcgac ggcgatgggc ttcacgtccg accgcgttgg

5821 gcacctggaa tcggtgtcgc tgctgcaccg cttccgcgtc ctggaccgtg gcaagaaaac

5881 gtcccgttgc caggtcctga tcgacgagga aatcgtcgtg ctgtttgctg gcgaccacta

5941 cacgaaattc atatgggaga agtaccgcaa gctgtcgccg acggcccgac ggatgttcga

6001 ctatttcagc tcgcaccggg agccgtaccc gctcaagctg gaaaccttcc gcctcatgtg

6061 cggatcggat tccacccgcg tgaagaagtg gcgcgagcag gtcggcgaag cctgcgaaga

6121 gttgcgaggc agcggcctgg tggaacacgc ctgggtcaat gatgacctgg tgcattgcaa

6181 acgctagggc cttgtggggt cagttccggc tggatctgct ctcccgctga cgccgtcccg

6241 gactgatggg ctgcctgtat cgagtggtga ttttgtgccg agctgccggt cggggagctg

6301 ttggctggct ggtggcagga tatattgtgg tgtaaa

//

**pRF-HU2**

LOCUS pRF-HU2 6323 bp DNA circular 11-JUL-2007

DEFINITION For targeted gene replacement.

SOURCE

ORGANISM

COMMENT The vector can be use for One-Step construction of vectors for targeted gene

replacement in filamentous fungi.

The vector has been designed for the simultaneous directional cloning of two PCR

amplicons into two unique sites of the vector, located on either site of the hph

(hygromycin B resistance gene), utilizing the USER friendly cloning technology

from NEB.

Add the following 5' overhangs to the primers for amplification of the two gene

specific homologous recombination flanks:

Gene-O1 (LB forward) 5´- GGTCTTAAU - Forward primer sequence

Gene-O2 (LB reverse) 5'- GGCATTAAU - Reverse primer sequence

Gene-A3 (RB forward) 5'- GGACTTAAU - Forward primer sequence

Gene-A4 (RB reverse) 5'- GGGTTTAAU - Reverse primer sequence

Digestion of vector prior to cloning:

1. Digest overnight with PacI

2. Digest for 2 hours with Nt.BbvCI (nicking enzyme)

3. Purify the vector DNA

Transformation of the vector into the fungi:

The vector is originate designed for Agrobacterium tumefaciens mediated

transformation, but can also be used for protoplast transformation.

COMMENT ORIGDB|EMBL

COMMENT AUTHORNAME|Rasmus J.N. Frandsen|

COMMENT AUTHOREML|raf@life.ku.dk|

COMMENT REPLTYPE|Plasmid

COMMENT EXTCHREPL|Bacteria

FEATURES Location/Qualifiers

promoter 313..677

/label=pTrpC

misc_structure 2858..2880

/label=RB

terminator 1727..2443

/label=TtrpC

CDS 678..1723

/label=hph

CDS 3932..4727

/label=KanR

CDS 5026..6175

/label=TrfA

misc_structure 6302..6323

misc_feature 2630..2653

/label=RF-2

misc_feature 2755..2780

/label=UCS\(RB)

misc_feature 266..291

/label=UCS\(LB)

primer_bind complement(400..423)

/label=RF-1

rep_origin 3028..3645

/label=oriV

BASE COUNT 1501 a 1609 c 1787 g 1426 t

ORIGIN

1 caaattgacg cttagacaac ttaataacac attgcggacg tttttaatgt actggggtgg

61 tttttctttt caccagtgag acgggcaaca gcggcgccat tcgccattca ggctgcgcaa

121 ctgttgggaa gggcgatcgg tgcgggcctc ttcgctatta cgccagctgg cgaaaggggg

181 atgtgctgca aggcgattaa gttgggtaac gccagggttt tcccagtcac gacgttgtaa

241 aacgacggcc agtgaattcg agctcgctga gggtttaatt aagtcctcag cgggcccatc

301 gatgatcagg cctcgacaga agatgatatt gaaggagcac tttttgggct tggctggagc

361 tagtggaggt caacaatgaa tgcctatttt ggtttagtcg tccaggcggt gagcacaaaa

421 tttgtgtcgt ttgacaagat ggttcattta ggcaactggt cagatcagcc ccacttgtag

481 cagtagcggc ggcgctcgaa gtgtgactct tattagcaga caggaacgag gacattatta

541 tcatctgctg cttggtgcac gataacttgg tgcgtttgtc aagcaaggta agtgaacgac

601 ccggtcatac cttcttaagt tcgcccttcc tccctttatt tcagattcaa tctgacttac

661 ctattctacc caagcatcga tatgaaaaag cctgaactca ccgcgacgtc tgtcgagaag

721 tttctgatcg aaaagttcga cagcgtctcc gacctgatgc agctctcgga gggcgaagaa

781 tctcgtgctt tcagcttcga tgtaggaggg cgtggatatg tcctgcgggt aaatagctgc

841 gccgatggtt tctacaaaga tcgttatgtt tatcggcact ttgcatcggc cgcgctcccg

901 attccggaag tgcttgacat tggggaattc agcgagagcc tgacctattg catctcccgc

961 cgtgcacagg gtgtcacgtt gcaagacctg cctgaaaccg aactgcccgc tgttctgcag

1021 ccggtcgcgg aggccatgga tgcgatcgct gcggccgatc ttagccagac gagcgggttc

1081 ggcccattcg gaccgcaagg aatcggtcaa tacactacat ggcgtgattt catatgcgcg

1141 attgctgatc cccatgtgta tcactggcaa actgtgatgg acgacaccgt cagtgcgtcc

1201 gtcgcgcagg ctctcgatga gctgatgctt tgggccgagg actgccccga agtccggcac

1261 ctcgtgcacg cggatttcgg ctccaacaat gtcctgacgg acaatggccg cataacagcg

1321 gtcattgact ggagcgaggc gatgttcggg gattcccaat acgaggtcgc caacatcttc

1381 ttctggaggc cgtggttggc ttgtatggag cagcagacgc gctacttcga gcggaggcat

1441 ccggagcttg caggatcgcc gcggctccgg gcgtatatgc tccgcattgg tcttgaccaa

1501 ctctatcaga gcttggttga cggcaatttc gatgatgcag cttgggcgca gggtcgatgc

1561 gacgcaatcg tccgatccgg agccgggact gtcgggcgta cacaaatcgc ccgcagaagc

1621 gcggccgtct ggaccgatgg ctgtgtagaa gtactcgccg atagtggaaa ccgacgcccc

1681 agcactcgtc cgagggcaaa ggaatagagt agatgccgac cgggatccac ttaacgttac

1741 tgaaatcatc aaacagcttg acgaatctgg atataagatc gttggtgtcg atgtcagctc

1801 cggagttgag acaaatggtg ttcaggatct cgataagata cgttcatttg tccaagcagc

1861 aaagagtgcc ttctagtgat ttaatagctc catgtcaaca agaataaaac gcgtttcggg

1921 tttacctctt ccagatacag ctcatctgca atgcattaat gcattggacc tcgcaaccct

1981 agtacgccct tcaggctccg gcgaagcaga agaatagctt agcagagtct attttcattt

2041 tcgggagacg agatcaagca gatcaacggt cgtcaagaga cctacgagac tgaggaatcc

2101 gctcttggct ccacgcgact atatatttgt ctctaattgt actttgacat gctcctcttc

2161 tttactctga tagcttgact atgaaaattc cgtcaccagc ccctgggttc gcaaagataa

2221 ttgcactgtt tcttccttga actctcaagc ctacaggaca cacattcatc gtaggtataa

2281 acctcgaaaa tcattcctac taagatgggt atacaatagt aaccatggtt gcctagtgaa

2341 tgctccgtaa cacccaatac gccggccgaa acttttttac aactctccta tgagtcgttt

2401 acccagaatg cacaggtaca cttgtttaga ggtaatcctt ctttctagag gatcctctac

2461 gccggacgca tcgtggccgg catcaccggc gccacaggtg cggttgctgg cgcctatatc

2521 gccgacatca ccgatgggga agatcgggct cgccacttcg ggctcatgag cgcttgtttc

2581 ggcgtgggta tggtggcagg ccccgtggcc gggggactgt tgggcgccat ctccttgcat

2641 gcaccattcc ttgcggcggc ggtgctcaac ggcctcaacc tactactggg ctgcttccta

2701 atgcaggagt cgcataaggg agagcgtcga gatctagagg atcccccgac tagtgctgag

2761 gcattaatta agacctcagc aagcttcgtg actcccttaa ttctccgctc atgatcagat

2821 tgtcgtttcc cgccttcagt ttaaactatc agtgtttgac aggatatatt ggcgggtaaa

2881 cctaagagaa aagagcgttt attagaataa tcggatattt aaaagggcgt gaaaaggttt

2941 atccgttcgt ccatttgttt gttcatgcca accacagggt tccagatccg acgagcaagg

3001 caagaccgag cgcctttgcg acgctcaccg ggctggttgc cctcgccgct gggctggcgg

3061 ccgtctatgg ccctgcaaac gcgccagaaa cgccgtcgaa gccgtgtgcg agacaccgcg

3121 gccgccggcg ttgtggatac ctcgcggaaa acttggccct cactgacaga tgaggggcgg

3181 acgttgacac ttgaggggcc gactcacccg gcgcggcgtt gacagatgag gggcaggctc

3241 gatttcggcc ggcgacgtgg agctggccag cctcgcaaat cggcgaaaac gcctgatttt

3301 acgcgagttt cccacagatg atgtggacaa gcctggggat aagtgccctg cggtattgac

3361 acttgagggg cgcgactact gacagatgag gggcgcgatc cttgacactt gaggggcaga

3421 gtgctgacag atgaggggcg cacctattga catttgaggg gctgtccaca ggcagaaaat

3481 ccagcatttg caagggtttc cgcccgtttt tcggccaccg ctaacctgtc ttttaacctg

3541 cttttaaacc aatatttata aaccttgttt ttaaccaggg ctgcgccctg tgcgcgtgac

3601 cgcgcacgcc gaaggggggt gccccccctt ctcgaaccct cccggcccgc taacgcgggc

3661 ctcccatccc cccaggcgta cgccactgga gcacctcaaa aacaccatca tacactaaat

3721 cagtaagttg gcagcatcac ccataattgt ggtttcaaaa tcggctccgt cgatactatg

3781 ttatacgcca actttgaaaa caactttgaa aaagctgttt tctggtattt aaggttttag

3841 aatgcaagga acagtgaatt ggagttcgtc ttgttataat tagcttcttg gggtatcttt

3901 aaatactgta gaaaagagga aggaaataat aaatggctaa aatgagaata tcaccggaat

3961 tgaaaaaact gatcgaaaaa taccgctgcg taaaagatac ggaaggaatg tctcctgcta

4021 aggtatataa gctggtggga gaaaatgaaa acctatattt aaaaatgacg gacagccggt

4081 ataaagggac cacctatgat gtggaacggg aaaaggacat gatgctatgg ctggaaggaa

4141 agctgcctgt tccaaaggtc ctgcactttg aacggcatga tggctggagc aatctgctca

4201 tgagtgaggc cgatggcgtc ctttgctcgg aagagtatga agatgaacaa agccctgaaa

4261 agattatcga gctgtatgcg gagtgcatca ggctctttca ctccatcgac atatcggatt

4321 gtccctatac gaatagctta gacagccgct tagccgaatt ggattactta ctgaataacg

4381 atctggccga tgtggattgc gaaaactggg aagaagacac tccatttaaa gatccgcgcg

4441 agctgtatga ttttttaaag acggaaaagc ccgaagagga acttgtcttt tcccacggcg

4501 acctgggaga cagcaacatc tttgtgaaag atggcaaagt aagtggcttt attgatcttg

4561 ggagaagcgg cagggcggac aagtggtatg acattgcctt ctgcgtccgg tcgatcaggg

4621 aggatatcgg ggaagaacag tatgtcgagc tattttttga cttactgggg atcaagcctg

4681 attgggagaa aataaaatat tatattttac tggatgaatt gttttagtac ctagatgtgg

4741 cgcaacgatg ccggcgacaa gcaggagcgc accgacttct tccgcatcaa gtgttttggc

4801 tctcaggccg aggcccacgg caagtatttg ggcaaggggt cgctggtatt cgtgcagggc

4861 aagattcgga ataccaagta cgagaaggac ggccagacgg tctacgggac cgacttcatt

4921 gccgataagg tggattatct ggacaccaag gcaccaggcg ggtcaaatca ggaataaggg

4981 cacattgccc cggcgtgagt cggggcaatc ccgcaaggag ggtgaatgaa tcggacgttt

5041 gaccggaagg catacaggca agaactgatc gacgcggggt tttccgccga ggatgccgaa

5101 accatcgcaa gccgcaccgt catgcgtgcg ccccgcgaaa ccttccagtc cgtcggctcg

5161 atggtccagc aagctacggc caagatcgag cgcgacagcg tgcaactggc tccccctgcc

5221 ctgcccgcgc catcggccgc cgtggagcgt tcgcgtcgtc tcgaacagga ggcggcaggt

5281 ttggcgaagt cgatgaccat cgacacgcga ggaactatga cgaccaagaa gcgaaaaacc

5341 gccggcgagg acctggcaaa acaggtcagc gaggccaagc aggccgcgtt gctgaaacac

5401 acgaagcagc agatcaagga aatgcagctt tccttgttcg atattgcgcc gtggccggac

5461 acgatgcgag cgatgccaaa cgacacggcc cgctctgccc tgttcaccac gcgcaacaag

5521 aaaatcccgc gcgaggcgct gcaaaacaag gtcattttcc acgtcaacaa ggacgtgaag

5581 atcacctaca ccggcgtcga gctgcgggcc gacgatgacg aactggtgtg gcagcaggtg

5641 ttggagtacg cgaagcgcac ccctatcggc gagccgatca ccttcacgtt ctacgagctt

5701 tgccaggacc tgggctggtc gatcaatggc cggtattaca cgaaggccga ggaatgcctg

5761 tcgcgcctac aggcgacggc gatgggcttc acgtccgacc gcgttgggca cctggaatcg

5821 gtgtcgctgc tgcaccgctt ccgcgtcctg gaccgtggca agaaaacgtc ccgttgccag

5881 gtcctgatcg acgaggaaat cgtcgtgctg tttgctggcg accactacac gaaattcata

5941 tgggagaagt accgcaagct gtcgccgacg gcccgacgga tgttcgacta tttcagctcg

6001 caccgggagc cgtacccgct caagctggaa accttccgcc tcatgtgcgg atcggattcc

6061 acccgcgtga agaagtggcg cgagcaggtc ggcgaagcct gcgaagagtt gcgaggcagc

6121 ggcctggtgg aacacgcctg ggtcaatgat gacctggtgc attgcaaacg ctagggcctt

6181 gtggggtcag ttccggctgg atctgctctc ccgctgacgc cgtcccggac tgatgggctg

6241 cctgtatcga gtggtgattt tgtgccgagc tgccggtcgg ggagctgttg gctggctggt

6301 ggcaggatat attgtggtgt aaa

//

**pRF-HUE**

LOCUS pRF-HUE 8709 bp DNA circular 11-JUL-2007

DEFINITION Vector for ectopic expression in filamentous fungi.

SOURCE

ORGANISM

COMMENT The vector can be used for ectopic over expression of genes in filamentous fungi.

The vector has been designed for the directional cloning of a single PCR

amplicon (typically a CDS plus terminator minus the start codon) after the

strong constitutive GAPDH promoter form Aspergillus nidulans, utilizing the

USER friendly cloning technology from NEB.

Add the following 5' overhangs to the primers for amplification of the coding

sequence including the native terminator:

Gene-O3 (LB forward) 5´- GGACTTAAU - Forward primer sequence

Gene-O4 (LB reverse) 5'- GGGTTTAAUG - Reverse primer sequence

The O4 primer contains a AUG (= ATG) which should be use as a start codon

in the gene you are trying to over express. Note that the vector does not contain

a 3'UTR or terminator, why these should be included in the PCR amplicon.

Digestion of vector prior to cloning:

1. Digest overnight with PacI

2. Digest for 2 hours with Nt.BbvCI (nicking enzyme)

3. Purify the vector DNA

Transformation of the vector into the fungi:

The vector is originate designed for Agrobacterium tumefaciens mediated

transformation, but can also be used for protoplast transformation.

COMMENT AUTHORNAME|Rasmus J.N. Frandsen|

COMMENT AUTHOREML|raf@life.ku.dk|

COMMENT REPLTYPE|Plasmid

COMMENT EXTCHREPL|Bacteria

FEATURES Location/Qualifiers

promoter 2708..3072

/label=pTrpC

misc_structure 5266..5288

/label=RB

terminator 4122..4838

/label=TtrpC

CDS 3073..4118

/label=hph

CDS 6340..7135

/label=KanR

CDS 7434..8583

/label=TrfA

misc_structure 1..22

/label=LB

promoter complement(323..2624)

/label=PgdpA

misc_feature 294..319

/label=UCS\(LB)

rep_origin 5436..6053

/label=oriV

primer_bind complement(2795..2818)

/label=RF-1

primer_bind 5025..5048

/label=RF-2

primer_bind complement(548..573)

/label=RF-3

BASE COUNT 2094 a 2199 c 2409 g 2007 t

ORIGIN

1 gcaggatata ttgtggtgta aacaaattga cgcttagaca acttaataac acattgcgga

61 cgtttttaat gtactggggt ggtttttctt ttcaccagtg agacgggcaa cagcggcgcc

121 attcgccatt caggctgcgc aactgttggg aagggcgatc ggtgcgggcc tcttcgctat

181 tacgccagct ggcgaaaggg ggatgtgctg caaggcgatt aagttgggta acgccagggt

241 tttcccagtc acgacgttgt aaaacgacgg ccagtgaatt cgagctcggt accgctgagg

301 gtttaattaa gtcctcagcc ccgggtgatg tctgctcaag cggggtagct gttagtcaag

361 ctgcgatgaa gtgggaaagc tcgaactgaa aggttcaaag gaataaggga tgggaaggat

421 ggagtatgga tgtagcaaag tacttactta ggggaaataa aggttcttgg atgggaagat

481 gaatatactg aagatgggaa aagaaagaga aaagaaaaga gcagctggtg gggagagcag

541 gaaaatatgg caacaaatgt tggactgacg caacgacctt gtcaaccccg ccgacacacc

601 gggcggacag acggggcaaa gctgcctacc agggactgag ggacctcagc aggtcgagtg

661 cagagcaccg gatgggtcga ctgccagctt gtgttcccgg tctgcgccgc tggccagctc

721 ctgagcggcc tttccggttt catacaccgg gcaaagcagg agaggcacga tatttggacg

781 ccctacagat gccggatggg ccaattaggg agcttacgcg ccgggtactc gctctaccta

841 cttcggagaa ggtactatct cgtgaatctt ttaccagatc ggaagcaatt ggacttctgt

901 acctaggtta atggcatgct atttcgccga cggctataca cccctggctt cacattctcc

961 ttcgcttact gccggtgatt cgatgaagct ccatattctc cgatgatgca atagattctt

1021 ggtcaacgag gggcacacca gcctttccac ttcggggcgg aggggcggcc ggtcccggat

1081 taataatcat ccactgcacc tcagagccgc cagagctgtc tggccagtgg cttattactc

1141 agcccttctc tctgcgtccg tccgtctctc cgcatgccag aaagagtcac cggtcactgt

1201 acagagctca cgagttcgtc acatttttct acaaatggtg gaggcggcgg attttaggct

1261 caagtcatga ccctctgggt cactccagaa tcagctaggt caacgaataa ggatgattct

1321 ataggaagat ccaggcaccg gtcaaccatg atctggacag atttgggagc tcggtataag

1381 ctctccacct atcttattct gtatagttta ggcttaaagt ttatccagga gatgttgctg

1441 aagtcgattt gagtccactt cctcactggt agctatacga ctttgatggt cgttgtaggg

1501 gctgtattag gtctcgatca aacacaaata gaattaaatg gtactcgagt ccactgaagg

1561 tggcttctcc gtcttccgta gccgtgccga aatccttaca gcttgtgttg tgtgactttt

1621 ggttacgccg tctgactttt gtggtgagct aactagagat catgctatat ctcctgattt

1681 aatacaatgc tcatcataac attccacctg gaactgctag caacgtttga cttgcattgt

1741 gcaacgccct ttgcagagct atcggatgat caatagtgcc acgttctaaa ttcaaccaac

1801 gcaggtgccc caagccttcg acatccggat gtatttcgaa aacctcatgg cgattgcagt

1861 cctcagattc atgttcattc caatgctcat tggtgaataa aaggttcaca gggaataagt

1921 tcaaactcga gatacttgag aatattgaaa gccaaaggac cctctatgct ccaagctaga

1981 gtctcagcct ggaaagcaaa tccaaatgaa gctatgctac ctccaattcc tcatcatctt

2041 atctataata cagagtcgaa gaatatcctc ttgacaccgc tccgtcctcc gacttcaata

2101 aggagcttac tcctccttga caccacccct ccagttcttc tcggcgttct ggagggaggc

2161 cttgtcggtc ttgggctggc cctggctgag aaagctgttg gcagccttaa agggacgctg

2221 gaggtcacca gtcgctggct tcccgaagac gtggatctta accagattcg aaagcgcctt

2281 cagcggatga tcgactggat cagaagagcg ttggtgtact tgaagtacag atgcatgacg

2341 gccatcatgc caacgcccat gaactggctc ttaatgagct ggcggaactg gcccttatcg

2401 tactccatgt tggtagttgt gacaggacga ggctcctcgc cgcttccaag cggagcaggc

2461 tcgacgtatt tcagtgtcga aagatctgca gagattactt caagtcagcc aactgcaaac

2521 agaatatccc gccaatagct ttgggacgat gcaagatata aacgaaaaag acgaaccgtt

2581 cttcttattg atttgagcct gtgtgtagag atacaaggga attcgggctg gccacggccg

2641 cctaggcgcg caaggatcct ctagatctcg aggcctgatc atcgatgggc ccatcgatga

2701 tcaggcctcg acagaagatg atattgaagg agcacttttt gggcttggct ggagctagtg

2761 gaggtcaaca atgaatgcct attttggttt agtcgtccag gcggtgagca caaaatttgt

2821 gtcgtttgac aagatggttc atttaggcaa ctggtcagat cagccccact tgtagcagta

2881 gcggcggcgc tcgaagtgtg actcttatta gcagacagga acgaggacat tattatcatc

2941 tgctgcttgg tgcacgataa cttggtgcgt ttgtcaagca aggtaagtga acgacccggt

3001 cataccttct taagttcgcc cttcctccct ttatttcaga ttcaatctga cttacctatt

3061 ctacccaagc atcgatatga aaaagcctga actcaccgcg acgtctgtcg agaagtttct

3121 gatcgaaaag ttcgacagcg tctccgacct gatgcagctc tcggagggcg aagaatctcg

3181 tgctttcagc ttcgatgtag gagggcgtgg atatgtcctg cgggtaaata gctgcgccga

3241 tggtttctac aaagatcgtt atgtttatcg gcactttgca tcggccgcgc tcccgattcc

3301 ggaagtgctt gacattgggg aattcagcga gagcctgacc tattgcatct cccgccgtgc

3361 acagggtgtc acgttgcaag acctgcctga aaccgaactg cccgctgttc tgcagccggt

3421 cgcggaggcc atggatgcga tcgctgcggc cgatcttagc cagacgagcg ggttcggccc

3481 attcggaccg caaggaatcg gtcaatacac tacatggcgt gatttcatat gcgcgattgc

3541 tgatccccat gtgtatcact ggcaaactgt gatggacgac accgtcagtg cgtccgtcgc

3601 gcaggctctc gatgagctga tgctttgggc cgaggactgc cccgaagtcc ggcacctcgt

3661 gcacgcggat ttcggctcca acaatgtcct gacggacaat ggccgcataa cagcggtcat

3721 tgactggagc gaggcgatgt tcggggattc ccaatacgag gtcgccaaca tcttcttctg

3781 gaggccgtgg ttggcttgta tggagcagca gacgcgctac ttcgagcgga ggcatccgga

3841 gcttgcagga tcgccgcggc tccgggcgta tatgctccgc attggtcttg accaactcta

3901 tcagagcttg gttgacggca atttcgatga tgcagcttgg gcgcagggtc gatgcgacgc

3961 aatcgtccga tccggagccg ggactgtcgg gcgtacacaa atcgcccgca gaagcgcggc

4021 cgtctggacc gatggctgtg tagaagtact cgccgatagt ggaaaccgac gccccagcac

4081 tcgtccgagg gcaaaggaat agagtagatg ccgaccggga tccacttaac gttactgaaa

4141 tcatcaaaca gcttgacgaa tctggatata agatcgttgg tgtcgatgtc agctccggag

4201 ttgagacaaa tggtgttcag gatctcgata agatacgttc atttgtccaa gcagcaaaga

4261 gtgccttcta gtgatttaat agctccatgt caacaagaat aaaacgcgtt tcgggtttac

4321 ctcttccaga tacagctcat ctgcaatgca ttaatgcatt ggacctcgca accctagtac

4381 gcccttcagg ctccggcgaa gcagaagaat agcttagcag agtctatttt cattttcggg

4441 agacgagatc aagcagatca acggtcgtca agagacctac gagactgagg aatccgctct

4501 tggctccacg cgactatata tttgtctcta attgtacttt gacatgctcc tcttctttac

4561 tctgatagct tgactatgaa aattccgtca ccagcccctg ggttcgcaaa gataattgca

4621 ctgtttcttc cttgaactct caagcctaca ggacacacat tcatcgtagg tataaacctc

4681 gaaaatcatt cctactaaga tgggtataca atagtaacca tggttgccta gtgaatgctc

4741 cgtaacaccc aatacgccgg ccgaaacttt tttacaactc tcctatgagt cgtttaccca

4801 gaatgcacag gtacacttgt ttagaggtaa tccttctttc tagaggatcc tctacgccgg

4861 acgcatcgtg gccggcatca ccggcgccac aggtgcggtt gctggcgcct atatcgccga

4921 catcaccgat ggggaagatc gggctcgcca cttcgggctc atgagcgctt gtttcggcgt

4981 gggtatggtg gcaggccccg tggccggggg actgttgggc gccatctcct tgcatgcacc

5041 attccttgcg gcggcggtgc tcaacggcct caacctacta ctgggctgct tcctaatgca

5101 ggagtcgcat aagggagagc gtcgagatct agaggatccc ccgactagtg cgcgatcgcg

5161 gccggccggc gcgccgttta aacggattta gcttcgtgac tcccttaatt ctccgctcat

5221 gatcagattg tcgtttcccg ccttcagttt aaactatcag tgtttgacag gatatattgg

5281 cgggtaaacc taagagaaaa gagcgtttat tagaataatc ggatatttaa aagggcgtga

5341 aaaggtttat ccgttcgtcc atttgtttgt tcatgccaac cacagggttc cagatccgac

5401 gagcaaggca agaccgagcg cctttgcgac gctcaccggg ctggttgccc tcgccgctgg

5461 gctggcggcc gtctatggcc ctgcaaacgc gccagaaacg ccgtcgaagc cgtgtgcgag

5521 acaccgcggc cgccggcgtt gtggatacct cgcggaaaac ttggccctca ctgacagatg

5581 aggggcggac gttgacactt gaggggccga ctcacccggc gcggcgttga cagatgaggg

5641 gcaggctcga tttcggccgg cgacgtggag ctggccagcc tcgcaaatcg gcgaaaacgc

5701 ctgattttac gcgagtttcc cacagatgat gtggacaagc ctggggataa gtgccctgcg

5761 gtattgacac ttgaggggcg cgactactga cagatgaggg gcgcgatcct tgacacttga

5821 ggggcagagt gctgacagat gaggggcgca cctattgaca tttgaggggc tgtccacagg

5881 cagaaaatcc agcatttgca agggtttccg cccgtttttc ggccaccgct aacctgtctt

5941 ttaacctgct tttaaaccaa tatttataaa ccttgttttt aaccagggct gcgccctgtg

6001 cgcgtgaccg cgcacgccga aggggggtgc ccccccttct cgaaccctcc cggcccgcta

6061 acgcgggcct cccatccccc caggcgtacg ccactggagc acctcaaaaa caccatcata

6121 cactaaatca gtaagttggc agcatcaccc ataattgtgg tttcaaaatc ggctccgtcg

6181 atactatgtt atacgccaac tttgaaaaca actttgaaaa agctgttttc tggtatttaa

6241 ggttttagaa tgcaaggaac agtgaattgg agttcgtctt gttataatta gcttcttggg

6301 gtatctttaa atactgtaga aaagaggaag gaaataataa atggctaaaa tgagaatatc

6361 accggaattg aaaaaactga tcgaaaaata ccgctgcgta aaagatacgg aaggaatgtc

6421 tcctgctaag gtatataagc tggtgggaga aaatgaaaac ctatatttaa aaatgacgga

6481 cagccggtat aaagggacca cctatgatgt ggaacgggaa aaggacatga tgctatggct

6541 ggaaggaaag ctgcctgttc caaaggtcct gcactttgaa cggcatgatg gctggagcaa

6601 tctgctcatg agtgaggccg atggcgtcct ttgctcggaa gagtatgaag atgaacaaag

6661 ccctgaaaag attatcgagc tgtatgcgga gtgcatcagg ctctttcact ccatcgacat

6721 atcggattgt ccctatacga atagcttaga cagccgctta gccgaattgg attacttact

6781 gaataacgat ctggccgatg tggattgcga aaactgggaa gaagacactc catttaaaga

6841 tccgcgcgag ctgtatgatt ttttaaagac ggaaaagccc gaagaggaac ttgtcttttc

6901 ccacggcgac ctgggagaca gcaacatctt tgtgaaagat ggcaaagtaa gtggctttat

6961 tgatcttggg agaagcggca gggcggacaa gtggtatgac attgccttct gcgtccggtc

7021 gatcagggag gatatcgggg aagaacagta tgtcgagcta ttttttgact tactggggat

7081 caagcctgat tgggagaaaa taaaatatta tattttactg gatgaattgt tttagtacct

7141 agatgtggcg caacgatgcc ggcgacaagc aggagcgcac cgacttcttc cgcatcaagt

7201 gttttggctc tcaggccgag gcccacggca agtatttggg caaggggtcg ctggtattcg

7261 tgcagggcaa gattcggaat accaagtacg agaaggacgg ccagacggtc tacgggaccg

7321 acttcattgc cgataaggtg gattatctgg acaccaaggc accaggcggg tcaaatcagg

7381 aataagggca cattgccccg gcgtgagtcg gggcaatccc gcaaggaggg tgaatgaatc

7441 ggacgtttga ccggaaggca tacaggcaag aactgatcga cgcggggttt tccgccgagg

7501 atgccgaaac catcgcaagc cgcaccgtca tgcgtgcgcc ccgcgaaacc ttccagtccg

7561 tcggctcgat ggtccagcaa gctacggcca agatcgagcg cgacagcgtg caactggctc

7621 cccctgccct gcccgcgcca tcggccgccg tggagcgttc gcgtcgtctc gaacaggagg

7681 cggcaggttt ggcgaagtcg atgaccatcg acacgcgagg aactatgacg accaagaagc

7741 gaaaaaccgc cggcgaggac ctggcaaaac aggtcagcga ggccaagcag gccgcgttgc

7801 tgaaacacac gaagcagcag atcaaggaaa tgcagctttc cttgttcgat attgcgccgt

7861 ggccggacac gatgcgagcg atgccaaacg acacggcccg ctctgccctg ttcaccacgc

7921 gcaacaagaa aatcccgcgc gaggcgctgc aaaacaaggt cattttccac gtcaacaagg

7981 acgtgaagat cacctacacc ggcgtcgagc tgcgggccga cgatgacgaa ctggtgtggc

8041 agcaggtgtt ggagtacgcg aagcgcaccc ctatcggcga gccgatcacc ttcacgttct

8101 acgagctttg ccaggacctg ggctggtcga tcaatggccg gtattacacg aaggccgagg

8161 aatgcctgtc gcgcctacag gcgacggcga tgggcttcac gtccgaccgc gttgggcacc

8221 tggaatcggt gtcgctgctg caccgcttcc gcgtcctgga ccgtggcaag aaaacgtccc

8281 gttgccaggt cctgatcgac gaggaaatcg tcgtgctgtt tgctggcgac cactacacga

8341 aattcatatg ggagaagtac cgcaagctgt cgccgacggc ccgacggatg ttcgactatt

8401 tcagctcgca ccgggagccg tacccgctca agctggaaac cttccgcctc atgtgcggat

8461 cggattccac ccgcgtgaag aagtggcgcg agcaggtcgg cgaagcctgc gaagagttgc

8521 gaggcagcgg cctggtggaa cacgcctggg tcaatgatga cctggtgcat tgcaaacgct

8581 agggccttgt ggggtcagtt ccggctggat ctgctctccc gctgacgccg tcccggactg

8641 atgggctgcc tgtatcgagt ggtgattttg tgccgagctg ccggtcgggg agctgttggc

8701 tggctggtg

//

**pRF-HU2E**

LOCUS pRF-HU2E 8696 bp DNA circular 11-JUL-2007

DEFINITION For targeted (in locus) over expression in filamentous fungi.

SOURCE

ORGANISM

COMMENT The vector has been designed for targeted (in locus) over expression of gene in

filamentous fungi. Compared to pRF-HU2 this vector contains the gpdA

promoter from Aspergillus nidulans.

Like pRF-HU2 this vector allows for the simultaneous directional cloning of two

PCR amplicons into two unique sites of the vector, located on either site of the

hph (hygromycin B resistance gene) and pGAPDH, utilizing the USER friendly

cloning technology from NEB.

Add the following 5' overhangs to the primers for amplification of the two gene

specific homologous recombination flanks:

Gene-O1 (LB forward) 5´- GGTCTTAAU - Forward primer sequence

Gene-O2 (LB reverse) 5'- GGCATTAAUG - Reverse primer sequence

Gene-O3 (RB forward) 5'- GGACTTAAU - Forward primer sequence

Gene-O4 (RB reverse) 5'- GGGTTTAAU - Reverse primer sequence

The O3 primer contains a AUG (= ATG) which can be used as start codon for

the gene you want to over express.

Digestion of vector prior to cloning:

1. Digest overnight with PacI

2. Digest for 2 hours with Nt.BbvCI (nicking enzyme)

3. Purify the vector DNA

Transformation of the vector into the fungi:

The vector is originate designed for Agrobacterium tumefaciens mediated

transformation, but can also be used for protoplast transformation.

COMMENT AUTHORNAME|Rasmus J.N. Frandsen|

COMMENT AUTHOREML|raf@life.ku.dk|

COMMENT REPLTYPE|Plasmid

COMMENT EXTCHREPL|Bacteria

FEATURES Location/Qualifiers

promoter 2708..3072

/label=pTrpC

misc_structure 5253..5275

/label=RB

terminator 4122..4838

/label=TtrpC

CDS 3073..4118

/label=hph

CDS 6327..7122

/label=KanR

misc_structure 7421..8570

/label=TrfA

misc_structure 1..22

/label=LB

misc_feature complement(323..2624)

/label=PgdpA

misc_feature 294..319

/label=UCS\(LB)

misc_feature 5150..5175

/label=UCS\(RB)

primer_bind complement(2795..2818)

/label=RF-1

primer_bind 5025..5048

/label=RF-2

primer_bind complement(548..573)

/label=RF-3

rep_origin 5423..6040

/label=oriV

BASE COUNT 2098 a 2192 c 2400 g 2006 t

ORIGIN

1 gcaggatata ttgtggtgta aacaaattga cgcttagaca acttaataac acattgcgga

61 cgtttttaat gtactggggt ggtttttctt ttcaccagtg agacgggcaa cagcggcgcc

121 attcgccatt caggctgcgc aactgttggg aagggcgatc ggtgcgggcc tcttcgctat

181 tacgccagct ggcgaaaggg ggatgtgctg caaggcgatt aagttgggta acgccagggt

241 tttcccagtc acgacgttgt aaaacgacgg ccagtgaatt cgagctcggt accgctgagg

301 gtttaattaa gtcctcagcc ccgggtgatg tctgctcaag cggggtagct gttagtcaag

361 ctgcgatgaa gtgggaaagc tcgaactgaa aggttcaaag gaataaggga tgggaaggat

421 ggagtatgga tgtagcaaag tacttactta ggggaaataa aggttcttgg atgggaagat

481 gaatatactg aagatgggaa aagaaagaga aaagaaaaga gcagctggtg gggagagcag

541 gaaaatatgg caacaaatgt tggactgacg caacgacctt gtcaaccccg ccgacacacc

601 gggcggacag acggggcaaa gctgcctacc agggactgag ggacctcagc aggtcgagtg

661 cagagcaccg gatgggtcga ctgccagctt gtgttcccgg tctgcgccgc tggccagctc

721 ctgagcggcc tttccggttt catacaccgg gcaaagcagg agaggcacga tatttggacg

781 ccctacagat gccggatggg ccaattaggg agcttacgcg ccgggtactc gctctaccta

841 cttcggagaa ggtactatct cgtgaatctt ttaccagatc ggaagcaatt ggacttctgt

901 acctaggtta atggcatgct atttcgccga cggctataca cccctggctt cacattctcc

961 ttcgcttact gccggtgatt cgatgaagct ccatattctc cgatgatgca atagattctt

1021 ggtcaacgag gggcacacca gcctttccac ttcggggcgg aggggcggcc ggtcccggat

1081 taataatcat ccactgcacc tcagagccgc cagagctgtc tggccagtgg cttattactc

1141 agcccttctc tctgcgtccg tccgtctctc cgcatgccag aaagagtcac cggtcactgt

1201 acagagctca cgagttcgtc acatttttct acaaatggtg gaggcggcgg attttaggct

1261 caagtcatga ccctctgggt cactccagaa tcagctaggt caacgaataa ggatgattct

1321 ataggaagat ccaggcaccg gtcaaccatg atctggacag atttgggagc tcggtataag

1381 ctctccacct atcttattct gtatagttta ggcttaaagt ttatccagga gatgttgctg

1441 aagtcgattt gagtccactt cctcactggt agctatacga ctttgatggt cgttgtaggg

1501 gctgtattag gtctcgatca aacacaaata gaattaaatg gtactcgagt ccactgaagg

1561 tggcttctcc gtcttccgta gccgtgccga aatccttaca gcttgtgttg tgtgactttt

1621 ggttacgccg tctgactttt gtggtgagct aactagagat catgctatat ctcctgattt

1681 aatacaatgc tcatcataac attccacctg gaactgctag caacgtttga cttgcattgt

1741 gcaacgccct ttgcagagct atcggatgat caatagtgcc acgttctaaa ttcaaccaac

1801 gcaggtgccc caagccttcg acatccggat gtatttcgaa aacctcatgg cgattgcagt

1861 cctcagattc atgttcattc caatgctcat tggtgaataa aaggttcaca gggaataagt

1921 tcaaactcga gatacttgag aatattgaaa gccaaaggac cctctatgct ccaagctaga

1981 gtctcagcct ggaaagcaaa tccaaatgaa gctatgctac ctccaattcc tcatcatctt

2041 atctataata cagagtcgaa gaatatcctc ttgacaccgc tccgtcctcc gacttcaata

2101 aggagcttac tcctccttga caccacccct ccagttcttc tcggcgttct ggagggaggc

2161 cttgtcggtc ttgggctggc cctggctgag aaagctgttg gcagccttaa agggacgctg

2221 gaggtcacca gtcgctggct tcccgaagac gtggatctta accagattcg aaagcgcctt

2281 cagcggatga tcgactggat cagaagagcg ttggtgtact tgaagtacag atgcatgacg

2341 gccatcatgc caacgcccat gaactggctc ttaatgagct ggcggaactg gcccttatcg

2401 tactccatgt tggtagttgt gacaggacga ggctcctcgc cgcttccaag cggagcaggc

2461 tcgacgtatt tcagtgtcga aagatctgca gagattactt caagtcagcc aactgcaaac

2521 agaatatccc gccaatagct ttgggacgat gcaagatata aacgaaaaag acgaaccgtt

2581 cttcttattg atttgagcct gtgtgtagag atacaaggga attcgggctg gccacggccg

2641 cctaggcgcg caaggatcct ctagatctcg aggcctgatc atcgatgggc ccatcgatga

2701 tcaggcctcg acagaagatg atattgaagg agcacttttt gggcttggct ggagctagtg

2761 gaggtcaaca atgaatgcct attttggttt agtcgtccag gcggtgagca caaaatttgt

2821 gtcgtttgac aagatggttc atttaggcaa ctggtcagat cagccccact tgtagcagta

2881 gcggcggcgc tcgaagtgtg actcttatta gcagacagga acgaggacat tattatcatc

2941 tgctgcttgg tgcacgataa cttggtgcgt ttgtcaagca aggtaagtga acgacccggt

3001 cataccttct taagttcgcc cttcctccct ttatttcaga ttcaatctga cttacctatt

3061 ctacccaagc atcgatatga aaaagcctga actcaccgcg acgtctgtcg agaagtttct

3121 gatcgaaaag ttcgacagcg tctccgacct gatgcagctc tcggagggcg aagaatctcg

3181 tgctttcagc ttcgatgtag gagggcgtgg atatgtcctg cgggtaaata gctgcgccga

3241 tggtttctac aaagatcgtt atgtttatcg gcactttgca tcggccgcgc tcccgattcc

3301 ggaagtgctt gacattgggg aattcagcga gagcctgacc tattgcatct cccgccgtgc

3361 acagggtgtc acgttgcaag acctgcctga aaccgaactg cccgctgttc tgcagccggt

3421 cgcggaggcc atggatgcga tcgctgcggc cgatcttagc cagacgagcg ggttcggccc

3481 attcggaccg caaggaatcg gtcaatacac tacatggcgt gatttcatat gcgcgattgc

3541 tgatccccat gtgtatcact ggcaaactgt gatggacgac accgtcagtg cgtccgtcgc

3601 gcaggctctc gatgagctga tgctttgggc cgaggactgc cccgaagtcc ggcacctcgt

3661 gcacgcggat ttcggctcca acaatgtcct gacggacaat ggccgcataa cagcggtcat

3721 tgactggagc gaggcgatgt tcggggattc ccaatacgag gtcgccaaca tcttcttctg

3781 gaggccgtgg ttggcttgta tggagcagca gacgcgctac ttcgagcgga ggcatccgga

3841 gcttgcagga tcgccgcggc tccgggcgta tatgctccgc attggtcttg accaactcta

3901 tcagagcttg gttgacggca atttcgatga tgcagcttgg gcgcagggtc gatgcgacgc

3961 aatcgtccga tccggagccg ggactgtcgg gcgtacacaa atcgcccgca gaagcgcggc

4021 cgtctggacc gatggctgtg tagaagtact cgccgatagt ggaaaccgac gccccagcac

4081 tcgtccgagg gcaaaggaat agagtagatg ccgaccggga tccacttaac gttactgaaa

4141 tcatcaaaca gcttgacgaa tctggatata agatcgttgg tgtcgatgtc agctccggag

4201 ttgagacaaa tggtgttcag gatctcgata agatacgttc atttgtccaa gcagcaaaga

4261 gtgccttcta gtgatttaat agctccatgt caacaagaat aaaacgcgtt tcgggtttac

4321 ctcttccaga tacagctcat ctgcaatgca ttaatgcatt ggacctcgca accctagtac

4381 gcccttcagg ctccggcgaa gcagaagaat agcttagcag agtctatttt cattttcggg

4441 agacgagatc aagcagatca acggtcgtca agagacctac gagactgagg aatccgctct

4501 tggctccacg cgactatata tttgtctcta attgtacttt gacatgctcc tcttctttac

4561 tctgatagct tgactatgaa aattccgtca ccagcccctg ggttcgcaaa gataattgca

4621 ctgtttcttc cttgaactct caagcctaca ggacacacat tcatcgtagg tataaacctc

4681 gaaaatcatt cctactaaga tgggtataca atagtaacca tggttgccta gtgaatgctc

4741 cgtaacaccc aatacgccgg ccgaaacttt tttacaactc tcctatgagt cgtttaccca

4801 gaatgcacag gtacacttgt ttagaggtaa tccttctttc tagaggatcc tctacgccgg

4861 acgcatcgtg gccggcatca ccggcgccac aggtgcggtt gctggcgcct atatcgccga

4921 catcaccgat ggggaagatc gggctcgcca cttcgggctc atgagcgctt gtttcggcgt

4981 gggtatggtg gcaggccccg tggccggggg actgttgggc gccatctcct tgcatgcacc

5041 attccttgcg gcggcggtgc tcaacggcct caacctacta ctgggctgct tcctaatgca

5101 ggagtcgcat aagggagagc gtcgagatct agaggatccc ccgactagtg ctgaggcatt

5161 aattaagacc tcagcaagct tcgtgactcc cttaattctc cgctcatgat cagattgtcg

5221 tttcccgcct tcagtttaaa ctatcagtgt ttgacaggat atattggcgg gtaaacctaa

5281 gagaaaagag cgtttattag aataatcgga tatttaaaag ggcgtgaaaa ggtttatccg

5341 ttcgtccatt tgtttgttca tgccaaccac agggttccag atccgacgag caaggcaaga

5401 ccgagcgcct ttgcgacgct caccgggctg gttgccctcg ccgctgggct ggcggccgtc

5461 tatggccctg caaacgcgcc agaaacgccg tcgaagccgt gtgcgagaca ccgcggccgc

5521 cggcgttgtg gatacctcgc ggaaaacttg gccctcactg acagatgagg ggcggacgtt

5581 gacacttgag gggccgactc acccggcgcg gcgttgacag atgaggggca ggctcgattt

5641 cggccggcga cgtggagctg gccagcctcg caaatcggcg aaaacgcctg attttacgcg

5701 agtttcccac agatgatgtg gacaagcctg gggataagtg ccctgcggta ttgacacttg

5761 aggggcgcga ctactgacag atgaggggcg cgatccttga cacttgaggg gcagagtgct

5821 gacagatgag gggcgcacct attgacattt gaggggctgt ccacaggcag aaaatccagc

5881 atttgcaagg gtttccgccc gtttttcggc caccgctaac ctgtctttta acctgctttt

5941 aaaccaatat ttataaacct tgtttttaac cagggctgcg ccctgtgcgc gtgaccgcgc

6001 acgccgaagg ggggtgcccc cccttctcga accctcccgg cccgctaacg cgggcctccc

6061 atccccccag gcgtacgcca ctggagcacc tcaaaaacac catcatacac taaatcagta

6121 agttggcagc atcacccata attgtggttt caaaatcggc tccgtcgata ctatgttata

6181 cgccaacttt gaaaacaact ttgaaaaagc tgttttctgg tatttaaggt tttagaatgc

6241 aaggaacagt gaattggagt tcgtcttgtt ataattagct tcttggggta tctttaaata

6301 ctgtagaaaa gaggaaggaa ataataaatg gctaaaatga gaatatcacc ggaattgaaa

6361 aaactgatcg aaaaataccg ctgcgtaaaa gatacggaag gaatgtctcc tgctaaggta

6421 tataagctgg tgggagaaaa tgaaaaccta tatttaaaaa tgacggacag ccggtataaa

6481 gggaccacct atgatgtgga acgggaaaag gacatgatgc tatggctgga aggaaagctg

6541 cctgttccaa aggtcctgca ctttgaacgg catgatggct ggagcaatct gctcatgagt

6601 gaggccgatg gcgtcctttg ctcggaagag tatgaagatg aacaaagccc tgaaaagatt

6661 atcgagctgt atgcggagtg catcaggctc tttcactcca tcgacatatc ggattgtccc

6721 tatacgaata gcttagacag ccgcttagcc gaattggatt acttactgaa taacgatctg

6781 gccgatgtgg attgcgaaaa ctgggaagaa gacactccat ttaaagatcc gcgcgagctg

6841 tatgattttt taaagacgga aaagcccgaa gaggaacttg tcttttccca cggcgacctg

6901 ggagacagca acatctttgt gaaagatggc aaagtaagtg gctttattga tcttgggaga

6961 agcggcaggg cggacaagtg gtatgacatt gccttctgcg tccggtcgat cagggaggat

7021 atcggggaag aacagtatgt cgagctattt tttgacttac tggggatcaa gcctgattgg

7081 gagaaaataa aatattatat tttactggat gaattgtttt agtacctaga tgtggcgcaa

7141 cgatgccggc gacaagcagg agcgcaccga cttcttccgc atcaagtgtt ttggctctca

7201 ggccgaggcc cacggcaagt atttgggcaa ggggtcgctg gtattcgtgc agggcaagat

7261 tcggaatacc aagtacgaga aggacggcca gacggtctac gggaccgact tcattgccga

7321 taaggtggat tatctggaca ccaaggcacc aggcgggtca aatcaggaat aagggcacat

7381 tgccccggcg tgagtcgggg caatcccgca aggagggtga atgaatcgga cgtttgaccg

7441 gaaggcatac aggcaagaac tgatcgacgc ggggttttcc gccgaggatg ccgaaaccat

7501 cgcaagccgc accgtcatgc gtgcgccccg cgaaaccttc cagtccgtcg gctcgatggt

7561 ccagcaagct acggccaaga tcgagcgcga cagcgtgcaa ctggctcccc ctgccctgcc

7621 cgcgccatcg gccgccgtgg agcgttcgcg tcgtctcgaa caggaggcgg caggtttggc

7681 gaagtcgatg accatcgaca cgcgaggaac tatgacgacc aagaagcgaa aaaccgccgg

7741 cgaggacctg gcaaaacagg tcagcgaggc caagcaggcc gcgttgctga aacacacgaa

7801 gcagcagatc aaggaaatgc agctttcctt gttcgatatt gcgccgtggc cggacacgat

7861 gcgagcgatg ccaaacgaca cggcccgctc tgccctgttc accacgcgca acaagaaaat

7921 cccgcgcgag gcgctgcaaa acaaggtcat tttccacgtc aacaaggacg tgaagatcac

7981 ctacaccggc gtcgagctgc gggccgacga tgacgaactg gtgtggcagc aggtgttgga

8041 gtacgcgaag cgcaccccta tcggcgagcc gatcaccttc acgttctacg agctttgcca

8101 ggacctgggc tggtcgatca atggccggta ttacacgaag gccgaggaat gcctgtcgcg

8161 cctacaggcg acggcgatgg gcttcacgtc cgaccgcgtt gggcacctgg aatcggtgtc

8221 gctgctgcac cgcttccgcg tcctggaccg tggcaagaaa acgtcccgtt gccaggtcct

8281 gatcgacgag gaaatcgtcg tgctgtttgc tggcgaccac tacacgaaat tcatatggga

8341 gaagtaccgc aagctgtcgc cgacggcccg acggatgttc gactatttca gctcgcaccg

8401 ggagccgtac ccgctcaagc tggaaacctt ccgcctcatg tgcggatcgg attccacccg

8461 cgtgaagaag tggcgcgagc aggtcggcga agcctgcgaa gagttgcgag gcagcggcct

8521 ggtggaacac gcctgggtca atgatgacct ggtgcattgc aaacgctagg gccttgtggg

8581 gtcagttccg gctggatctg ctctcccgct gacgccgtcc cggactgatg ggctgcctgt

8641 atcgagtggt gattttgtgc cgagctgccg gtcggggagc tgttggctgg ctggtg

//
