# Supplementary material for: Efficient four fragment cloning for the construction of vectors for targeted gene replacement in filamentous fungi
Source: BMC Mol Biol. 2008 Aug 1;9:70. doi: 10.1186/1471-2199-9-70 (PMC2533011; doi:10.1186/1471-2199-9-70)
Supplement: Additional File 3 — Protocols for USER Friendly cloning. [file 1471-2199-9-70-S3.doc]

**Protocols for USER Friendly cloning**

Version 1.2 by Rasmus J.N. Frandsen

**Aim:**

Single step construction of vectors for targeted gene replacement in fungi

**Contents:**

1. Available vectors and design of primers
2. Preparation of vectors for USER Friendly cloning
3. The USER Friendly cloning reaction

1. Available vectors and design of primers

| **Vector name** | **Application** |
| --- | --- |
| **pRF-HU** | For USER friendly directional cloning of a single PCR amplicon.  The USER cloning site is located at the LB. |
| **pRF-HU2** | For USER friendly directional cloning of two PCR amplicons into two individual sites of the vector, located at LB and RB.  The vector can be used for single step construction of vectors for targeted gene replacement. |
| **pRF-HUE** | For USER friendly directional cloning of a single PCR amplicon and expression from the constitutive *gpdA* promoter from *Aspergillu*s *nidulans*  The vector can be used for heterologous or homologous ectopic expression of genes. Note that the vector does not contain a terminator, why the endogenic terminator from the cloned gene should be included in the PCR amplicon.  The USER cloning site is located at the LB. |
| **pRF-HU2E** | For USER friendly directional cloning of two PCR amplicons into two individual sites of the vector, located at LB and RB.  The vector can be used for single step construction of vectors for *in locus* overexpression driven by the *A. nidulans* gpdA promoter by homologous recombination (promoter replacement). |

**Location of USER cloning sites (UCS):**

pRF-HU RB – (tTrpC-*hph*(🡨)-pTrpC) – UCS – LB

pRF-HU2 RB – UCS – (tTrpC-*hph*(🡨)-pTrpC) – UCS – LB

pRF-HUE RB – (tTrpC-*hph*(🡨)-pTrpC) – (🡨) PgpdA – UCS – LB

pRF-HU2E RB – UCS – (tTrpC-*hph*(🡨)-pTrpC) – (🡨) PgpdA – UCS – LB

**Design of primers**

1. Use Vector NTI or another primer design program to design the primers for amplifying the two homologous recombination sequences.
2. Add the following 5’ overhangs to the designed primers. Optimally, include the 5’extensions in the primer property calculations.

Gene-O1 (RB reverse) 5’- GGTCTTAAU - Reverse primer sequence

Gene-O2 (RB forward) 5’- GGCATTAAU - Forward primer sequence

Gene-O3 (LB reverse) 5’- GGACTTAAU - Reverse primer sequence

Gene-O4 (LB forward) 5´- GGGTTTAAU - Forward primer sequence

1. Order the primers. Note that the 2-Deoxyuridine base is typically classified as a modification.

When working with the overexpression vectors (pRF-HUE and pRF-HU2E) you can take advantage of the AU found in the O3 primer. If you design your primers to amplify the CDS of the target gene, from the G in ATG (start codon) you will automatically get an AUG (=ATG) when adding the USER overhangs.

**The two USER cloning sites before and after digestion with *PacI* and *Nt.BbvCI***

**UCS at RB**

*PacI Nt.BbvCI*

| |

5’- GCTGA GGTCTTA AT|TAATGCC|TCAGC -3’

3’- CGACT|CCAGAAT|TA ATTACGG AGTCG -5’

| |

*Nt.BbvCI PacI*

5’- GCTGAGGTCTTAAT TCAGC -3’

3’- CGACT TAATTACGGAGTCG -5’

­­­­­­­­­­­­

**UCS at LB**

*PacI Nt.BbvCI*

| |

5’- GCTGA GGACTTA AT|TAAACCC|TCAGC -3’

3’- CGACT|CCTGAAT|TA ATTTGGG AGTCG -5’

| |

*Nt.BbvCI PacI*

5’- GCTGAGGACTTAAT TCAGC -3’

3’- CGACT TAATTTGGGAGTCG -5’

***2. Preparation of vectors for USER Friendly cloning***

**Production of pRF-HU2 and pRF-HU2E vectors:**

The pRF-HU2 and pRF-HU2E vectors are maintained as low copy number plasmids in *E. coli*. The protocol requires 10 µg, why it is necessary to maxiprep the vectors from 3 L of LB culture.

1. Inoculate a 10 ml LB + kanamycin A (25 μg/ml) starter culture with a single colony and incubate overnight at 37 ^o^C with 200 rpm stir.
2. Inoculate a 3 L of LB + kanamycin A (25 μg/ml) culture with 3 ml of overnight starter culture in the late afternoon and incubate overnight at 37 ^o^C with 200 rpm stir.
3. Harvest the culture with centrifugation at 6000 x g for 10 minutes at 4 ^o^C.

Remove the supernatant.

1. Purify the plasmid using a suitable technique.

We normally use the Qiagen ‘HiSpeed Plasmid Maxi Kit’ (Qiagen cat. no. 12662 or 12663), following the manufactures recommendations.

**Linearization of the vectors:**

1. Digest 10 µg pRF-HU2 or pRF-HU2E with 70 units (7 μl) *PacI* (New England Biolabs, Cat. no. R0547) overnight at 37 °C in a total volume of 300 µl with NEBuffer 4 + BSA.
2. The next day, add an additional 20 units (2 μl) of *PacI* and 40 units (4 μl) *Nt.BbvCI* (New England Biolabs, Cat. no. R0632), and incubate for 1 hour at 37 °C.
3. Verify the linearization of the vector by gel electrophoresis (2-3µl). NB. When you include the time to run the gel electrophoresis the total digestion time with *Nt.BbvCI* will amount to 1½ hour which is more than sufficient.
4. Purify the linearized vector using the ‘illustra GFX PCR DNA and Gel Band Purification kit’ from GE healthcare (Cat. no. 28-9034-70 or 28-9034-71). Use two mini spin columns to purify the 300 μl and elute the DNA in 50 μl of EB buffer.
5. Determine the concentration by gel electrophoresis and measure it on a spectrophotometer

This protocol normally results in a DNA concentration of app. 50 μg/μl and a volume of 90 μl, sufficient for 20 cloning reactions. Divide the app. 90 μl into four aliquots and store them at -20 ^o^C until use, this will prevent degradation of the DNA by repeated freezing and thawing rounds.

3. USER Friendly cloning reaction

1. Perform PCR with PfuTurbo® C_x_ Hotstart DNA polymerase (Stratagene) using the manufacturer’s recommendations in a reaction volume of 15 µl per reaction.

The PfuTurbo® Cx Hotstart DNA polymerase is currently the only commercially available proofreading DNA polymerase which can amplify uracil containing templates.

1. Check the success of the PCR reaction by loading 5 µl of the reaction volume on an agarose gel. Note that it is not necessary to purify the PCR amplicon before the USER Friendly cloning reaction.
2. Mix the following in a 0.2 ml PCR tube

PCR product 1 10 μl (150 ng)

PCR product 2 10 μl (150 ng)

Linearized vector 4 μl (200 ng)

USER enzyme mix 1 μl

Total volume ~ 25 μl

It is not necessary to add any buffer to the reaction as the USER enzymes can function in most 1 x DNA polymerase buffers. However, if you are forced to purify the PCR products due to many unspecific bands you should add DNA polymerase buffer to the reaction.

1. Incubate at 37 °C for 20 min followed by 25 °C for 20 min (we use a PCR cycler for this).
2. Transformation:
   1. Transfer the reaction mix to a precooled 1.5 ml Eppendorf tube.
   2. Add 50 μl of chemically competent *E. coli* JM109 cells (> 1*10^6^ cfu) to the Eppendorf tube.
   3. Mix gently by tapping on the tube a couple of times and incubate for 30 minutes on ice.
   4. Heat shock the cells by incubating them at 42 ^o^C for 45 seconds, immediately afterward return the cells to ice and incubate for 2 minutes.
   5. Add 450 μl SOC medium, mix by inverting the tubes a couple of times and incubate for 1 hour at 37 ^o^C with 300 rpm.
   6. Pellet the cells in a table top centrifuge (1 minute at 5.000 rpm), discard 9/10 of the supernatant and resuspend the cells by pipetting up and down until all cells have been separated. Plate the approximately 50 μl onto a single LB plate supplemented with 25 μg/ml kanamycin A. Incubate the plate overnight at 37 ^o^C.

**NB. Electrocompetent cells cannot be used as the electro shock will cause the hybridized DNA fragments to dissociate.**

1. The next day: Isolate the obtained colonies onto a new LB plate supplemented with 25 μg/ml kanamycin A and incubate the plate overnight at 37 ^o^C.
2. The next day: Screen 5-10 of the resulting colonies by PCR using the insert specific primers used to amplify the two inserts in step 1 (two reactions pr colony). It is also possible to perform the screening on the plates from step 6, however, we normally get a very high level of background due the free amplicon DNA found on the plates from the cloning reaction.
